# Steroid dynamics in myalgic encephalomyelitis / chronic fatigue syndrome: a case-control study using ultra performance supercritical fluid chromatography tandem mass spectrometry

**DOI:** 10.1186/s12967-025-06841-4

**Published:** 2025-07-25

**Authors:** Natalie Thomas, S. J. Kumari A. Ubhayasekera, Christopher W. Armstrong, Katherine Huang, Jonas Bergquist

**Affiliations:** 1 Department of Biochemistry and Pharmacology, Bio21 Molecular Science and Biotechnology Institute, University of Melbourne, Parkville, VIC 3010 Australia; 2 Analytical Chemistry and Neurochemistry, Department of Chemistry – BMC, Uppsala University, Box 599, Uppsala, 75124 Sweden; 3 The ME/CFS Collaborative Research Centre at Uppsala University, Uppsala, Sweden

**Keywords:** ME/CFS, Steroidomics, Ultra-performance supercritical fluid chromatography tandem mass spectrometry (UPSFC-MS/MS), Partial correlations, Network analysis, Neuroendocrine dysfunction, Hormone metabolism, Chronic illness, Homeostasis

## Abstract

**Background:**

Myalgic encephalomyelitis / chronic fatigue syndrome (ME/CFS) is a multisystem disorder characterised by unrelenting fatigue, post-exertional malaise, and dysfunction across immune, nervous, metabolism, and endocrine systems. Given the broad role of steroid hormones in regulating these systems, this study investigated differences in the steroid metabolome and network dynamics between ME/CFS patients and matched controls.

**Methods:**

Blood plasma steroid levels were quantified using Ultra-Performance Supercritical Fluid Chromatography- Tandem Mass Spectrometry (UPSFC-MS/MS) in ME/CFS patients (*n* = 24) and age and gender matched controls (*n* = 24). Group comparisons of absolute steroid concentrations were performed using Mann-Whitney U tests. Partial Spearman correlation networks were evaluated to examine direct associations between steroids within each group, and centrality metrics were used to evaluate structural differences. Steroid-steroid ratios were analysed to reflect biochemical relationships. Multivariate analysis with Orthogonal Partial Least Squares Discriminant Analysis (OPLS-DA) was also conducted.

**Results:**

No significant group differences in absolute steroid concentrations were observed following FDR correction. However, network analysis revealed a marked reduction in direct steroid-steroid relationships in ME/CFS, with controls exhibiting 52 significant partial correlations, while the ME/CFS group retained only one (cortisol - corticosterone). Centrality analysis further revealed a shift in network structure, with cortisone emerging as highly central in ME/CFS (degree = 7, betweenness = 16.7), despite being peripheral in controls, and progesterone showing reduced integration in ME/CFS (degree = 3 vs. 12, eigenvector = 0.40 vs. 0.93). Steroid-steroid ratio analysis revealed a higher cortisol-to-pregnanolone ratio and a lower pregnanolone-to-progesterone ratio in ME/CFS, although these findings did not remain significant after FDR correction. OPLS-DA indicated a modest relationship between steroid levels and group classification (R²Y = 22.8%), but negative Q² values suggested poor predictive power.

**Conclusions:**

Despite no significant differences in absolute steroid levels, network analysis revealed profound disruptions in steroid-steroid relationships in ME/CFS compared to controls, suggesting disrupted steroid homeostasis. Collectively the results suggest dysregulation of HPA axis function and progestogen pathways, as demonstrated by altered partial correlations, centrality profiles, and steroid ratios. These findings illustrate the importance of hormone network dynamics in ME/CFS pathophysiology and underscores the need for more research into steroid metabolism.

**Supplementary Information:**

The online version contains supplementary material available at 10.1186/s12967-025-06841-4.

## Background

Myalgic encephalomyelitis/chronic fatigue syndrome (ME/CFS) is a disabling clinical condition defined by persistent, unexplained fatigue that is unresponsive to rest. Post-exertional malaise (PEM), a hallmark feature of the illness that distinguishes it from other chronic illnesses, is experienced as the severe worsening of symptoms following physical or mental exertion which can last weeks or months and may lead to reduced baseline functioning [1]. This condition is further characterised by cognitive impairment, unrefreshing sleep, chronic pain, autonomic dysregulation, and disruptions in both neuroendocrine and immune function [2]. The impact of ME/CFS on daily life profoundly restricts routine activities and results in a lower quality of life than that associated with cancer, multiple sclerosis, or stroke [3]. Recognised by the World Health Organization (WHO) as a neurological disorder [4], ME/CFS is driven by disruptions to neuro-immune-metabolic-endocrine homeostasis that are believed to be central to its complex pathology [1].

ME/CFS affects individuals across all races, ages, and genders, yet being female is one of the most reliable risk factors for diagnosis, with a 3:1 female predominance [1]. This gender disparity appears to emerge after puberty (ages 10–19), with another diagnostic incidence peak during a women’s reproductive years, often coinciding with pregnancy and the postpartum period (ages 30–40). Pregnancy and perimenopause have been reported as a trigger of ME/CFS symptom onset, and women have reported ME/CFS symptom fluctuation with hormonal changes, including those associated with the menstrual cycle and perimenopause [5]. Accordingly, it can be suggested that such endocrine shifts can influence susceptibility to ME/CFS, with gonadal sex steroids driving such changes [6]. The importance of the endocrine system is further highlighted by extensive research on adrenal cortisol, one of the most widely studied biomarkers in ME/CFS [7–10], in addition to limited research showing changes in TS/ T4 thyroid hormones [11], growth hormone [12], and aldosterone steroids [13].

The main purpose of the endocrine system is to act as the body’s communication and adaption network, integrating signals from internal and external environments and orchestrating multi-system responses over time. Steroid and peptide hormones, regulated by the hypothalamic-pituitary (HP) endocrine axes, pervasively affect the nervous system, immune function, and energy metabolism, working as master regulators governing physiological homeostasis and coordinating fundamental body functions [14]. All steroid hormones are synthesised from the precursor cholesterol through a shared biosynthesis pathway, where the levels and activity of one hormone influences the others [15]. Once released by endocrine glands and other tissues including adipose tissue, skin, and the brain, they travel through the bloodstream to target organs and tissues, where they exert their effects by binding to specific receptors which are expressed across virtually all organs and tissues. Consequently, dysregulation within the HP axis can have far reaching consequences, leading to immune dysfunction, autonomic, metabolic imbalances, and neurological symptoms, all commonly observed in ME/CFS patients [16]. Investigating the network dynamics of these hormones, rather than examining each in isolation, provides a more integrated understanding of their biosynthesis, metabolism, and regulatory mechanisms. This network-based approach is essential to elucidate potential hormone imbalances, that may drive disease processes in ME/CFS.

Ultra-Performance Supercritical Fluid Chromatography Tandem Mass Spectrometry (UPSFC-MS/MS) is an emerging analytical technique that offers significant advantages for the analysis of steroid hormones and their metabolites [17]. Traditional immunoassays are limited due to their cross-reactivity and associated inaccurate quantification, especially with structurally similar steroids including progesterone and 17 α - hydroxyprogesterone and cortisol and cortisone [18–20], which prevents simultaneous analysis of multiple steroids in the same analytical run. Consequently, mass spectrometry (MS) techniques are now considered the gold standard for steroid quantification [21], with both gas chromatography (GC) and liquid chromatography offering distinct methodological strengths and limitations. UPSFC-MS combines the advantages of high resolution and specificity of GC with the high-throughput and efficiency capabilities of ultraperformance LC [22]. This enables accurate and simultaneous detection of low-abundance steroid metabolites in a single analytical run, making it a powerful tool for steroidomics research [17]. Simultaneously analysing steroids provides a comprehensive overview of hormone biosynthesis and interactions within the steroidome, which is critical for understanding the endocrine networks involved in a complex, heterogenous syndrome like ME/CFS.

### Broad aim

The broad aim of this study was to perform a comprehensive comparative analysis of the steroid metabolome and analyse network dynamics in ME/CFS versus non-ME/CFS control group to enable the investigation of endocrine dysregulation in ME/CFS. Steroids, including progestogens (progesterone (prog, P_4_), 17α-hydroxyprogesterone (17OHP), pregnenolone (P_5_), pregnanolone (PNL)), corticosteroids (aldosterone (Aldo), cortisol (F), corticosterone (B), 1-deoxycorticosterone (DOC), 11-deoxycortisol (11DOC), cortisone (Cot)), androgens (androstenedione (AED, androsterone (ADT, testosterone (T), etiocholanolone (Etn), dehydroepiandrosterone (DHEA)), and estrogens (estradiol (E_2_), estrone, (E_1_)) were profiled in peripheral plasma blood samples using ultra-performance supercritical fluid chromatography couple to tandem mass spectrometry (UPSFC-MS/MS), as previously reported [17]. A total of 48 participants (*n* = 24 ME/CFS, *n* = 24 controls), matched by age and gender, were included in the analysis. Gender is used throughout the manuscript, as the variable was defined by self-reported gender rather than sex.

## Methods

### Study population and sample collection

Study participants were diagnosed at the Gottfries clinic in Göteborg between years 2013–2018 after approval from the regional committee in Göteborg 2016:966–15. All methods and EDTA plasma sampling were carried out in accordance with relevant guidelines and regulations including the Declaration of Helsinki and a written consent was signed. All patients fulfilled the Canadian Consensus Criteria, the International Consensus Criteria and the IOM criteria (Carruthers et al., 2003; Carruthers et al., 2011; Institute of Medicine, 2015). Clinical symptom severity was assessed using two validated rating instruments. The Mental Fatigue Scale (0–44) evaluates mental fatigue over the past month based on 14 self-rated items, with higher scores indicating greater fatigue severity [23]. The FibroFatigue Scale (0–72) is an observer-rated tool comprising 12 items that assess fatigue-related multisystem symptom burden, including pain, sleep disturbance, and cognitive dysfunction [24]. The two most common co-morbidities in patients were Hypothyroidism and Fibromyalgia, but other diseases commonly seen in ME/CFS, such as Irritable Bowel Syndrome (IBS) were also present [25, 26]. When recorded, the two most common disease-triggering infections were the Flu and Upper respiratory infection in addition to infections of Neuroborreliosis, TWAR, Epstein Barr virus, Gastroenteritis and Meningitis. In addition to the patient samples, EDTA plasma samples were collected from 24 age and gender-matched controls from the blood bank in Uppsala Academic Hospital. All blood samples were collected from fasted participants in the AM.

### Extraction of steroids hormones

Sample preparation commenced with liquid-liquid extraction (LLE). In brief, 50 µL of plasma was mixed with the mixture of internal standards and steroids were extracted to 500 µL of tert-butyl methyl ether (MTBE). Samples were gently vortexed for 10 min and were centrifuged at 1000 g for 5 min. The supernatant was collected and the solvent was evaporated under a stream of nitrogen gas. During the extraction, the steroids were protected against oxidation by the addition of 0.05 mg/mL BHT to the extraction solvent (MTBE). Identification and Quantification of the steroids achieved by derivatization of extracted analytes with methoxyamine hydrochloride to form oxime derivative of steroids. Steroid-Oxime and were dissolved in 50 µL methanol prior to the analysis by UPSFC- MS/MS [15]. Steroid-oxime derivatives were analyzed using an Ultra-Performance Supercritical Fluid Chromatography Tandem Mass Spectrometry (UPSFC-MS/MS) system (Waters, USA). Separation was performed on an Acquity UPC² BEH column at 40 °C with a 2.0 mL/min flow rate using 0.1% formic acid in methanol: isopropanol (1:1) as the mobile phase. Steroids were eluted using a gradient program with a total run time of 5 min. Detection was conducted in positive electrospray ionization mode (ESI⁺) with multiple reaction monitoring (MRM) over an m/z range of 100–600. Nitrogen and argon were used as the desolvation and collision gases, respectively. The quantification was based on a multiple reaction monitoring (MRM) method and collision energy and scan dwell time were set according to Table 1. Quantification of steroids was performed using the corresponding isotopic internal standard Data were acquired and processed using MassLynx NT4.1 software, and quantification of steroids was performed using the corresponding isotopic internal standard [15].

**Table 1:** Demographic characteristics of study population

Category	*n*	Age (Mean)	Age (SD)
STUDY POPULATION	48	43	11.88
ME/CFS	24	43	12.22
CONTROL	24	43.1	11.79
Females (Total population)	32	46	12.33
Males (Total population)	16	37.1	8.49
Females (ME/CFS group)	16	46	12.78
Males (ME/CFS group)	8	36.9	8.79
Females (Control group)	16	46	12.27
Males (Control group)	8	37.4	8.78

ME/CFS; Myalgic Encephalomyelitis, SD; Standard deviation

### Statistical analysis

#### Absolute levels and multiple testing correction

Mann-Whitney U tests were performed to assess differences in the circulating levels of steroid hormones in ME/CFS patients compared to controls. Steroid level comparisons between the ME/CFS and CTRL cohorts were also stratified by gender. The significance threshold for all statistical tests were set at *p* < 0.05, with adjustments for multiple comparisons applied using the Benjamini-Hochberg (BH) False Discovery Rate (FDR) method. A q value threshold of < 0.3 was applied as a balanced, discovery orientated threshold to prioritise potentially meaningful steroid differences future validation. Cohen’s d was calculated as the standardised difference between group means where negative values indicate lower ME/CFS levels compared to controls). Power calculations were conducted using a two-sided independent samples t-test framework (α = 0.05, 80% power) to estimate the minimum sample size per group to detect a statistically significant difference, based effect sizes (Cohen’s d) observed in the dataset.

#### Correlation with clinical severity

Spearman correlations were performed between steroid levels and clinical severity scores (Mental Fatigue and FibroFatigue scales), with BH correction applied for multiple testing. Results are presented in Supplementary Table 1.

#### Steroid-steroid ratio comparison

Steroid-steroid ratios were compared using the Mann-Whitney U test to assess group differences. The use of steroid-steroid ratios can be beneficial to assess the balance or relationship between different hormones or substances within the body [27, 28].

#### Partial spearman correlations and concentration network analysis

Spearman correlations were calculated for all steroid pairs to assess overall relationships using transformed data. To address multicollinearity within steroid networks, partial spearman correlations were used to isolate direct relationships whilst controlling for the influence of other steroids. This network approach has been increasingly used to dissect complex biological systems, including endocrine disorders [29].

Partial correlations were calculated for all 17 measured steroids pairs within groups using the R package ppcor v1.1 [30]. To compare correlation coefficients for the same steroid pairs across the ME/CFS and CTRL groups (between groups), statistical significance was tested using the R package cocor v1.1.4 [31].

The spearman partial correlations were also visualised in concentration network plots (R package igraph v1.3.5) where each node represents a steroid, and each edge represents the spearman partial correlation between two steroid concentrations. Network visualisation employed the Fruchterman − Reingold algorithm, placing highly correlated nodes closer together and centrally in the network, whilst the nodes with weaker connections are more peripheral [32]. Further, centrality measures including degree centrality, betweenness centrality, closeness centrality, and eigenvector centrality, were calculated to assess the relative importance of each steroid within the correlation-based network for ME/CFS and control groups (R package igraph v1.3.5). All analyses were performed in RStudio.

#### Orthogonal partial least squares discriminant analysis

Orthogonal Partial Least Squares Discriminant Analysis (OPLS-DA) was employed to identify the variables that contribute most significantly to the differences between groups. OPLS-DA is particularly well-suited for high multicollinearity data [33]. All steroid variables were scaled to pareto scaling. Model validity was evaluated by parameters R2 and Q2, and Variable importance projection (VIP) scores of greater than 1.2 were identified as significant contributors to the model. OPLS-DA analysis was conducted using MetaboAnalyst 6.0 [34]. To reduce dimensionality and minimise the risk of overfitting in supervised modelling, we applied feature selection based on effect size. Specifically, we calculated Cohen’s d for each steroid and retained those with an absolute effect size greater than 0.3 (|d| >0.3) between ME/CFS and control groups for inclusion in the OPLS-DA model. This feature selection was performed on the full cohort only as stratified subgroup analyses (e.g. by gender) would result in insufficient sample sizes with likely risk of model instability.

#### Data preprocessing and transformation

Missing values were replaced with a lower limit of detection (LOD) of 0.05nmol/mg. Steroid data levels were log_10_-transformed with offset (log_10_(Y + 1), where Y is the steroid concentration, for all analyses except for absolute level comparisons. No outlier removal was implemented. Absolute steroid data were determined not to be normally distributed by a Shapiro-Wilk test and therefore nonparametric tests were employed.

## Results

### Two group comparison of absolute steroid levels

**Fig. 1:**
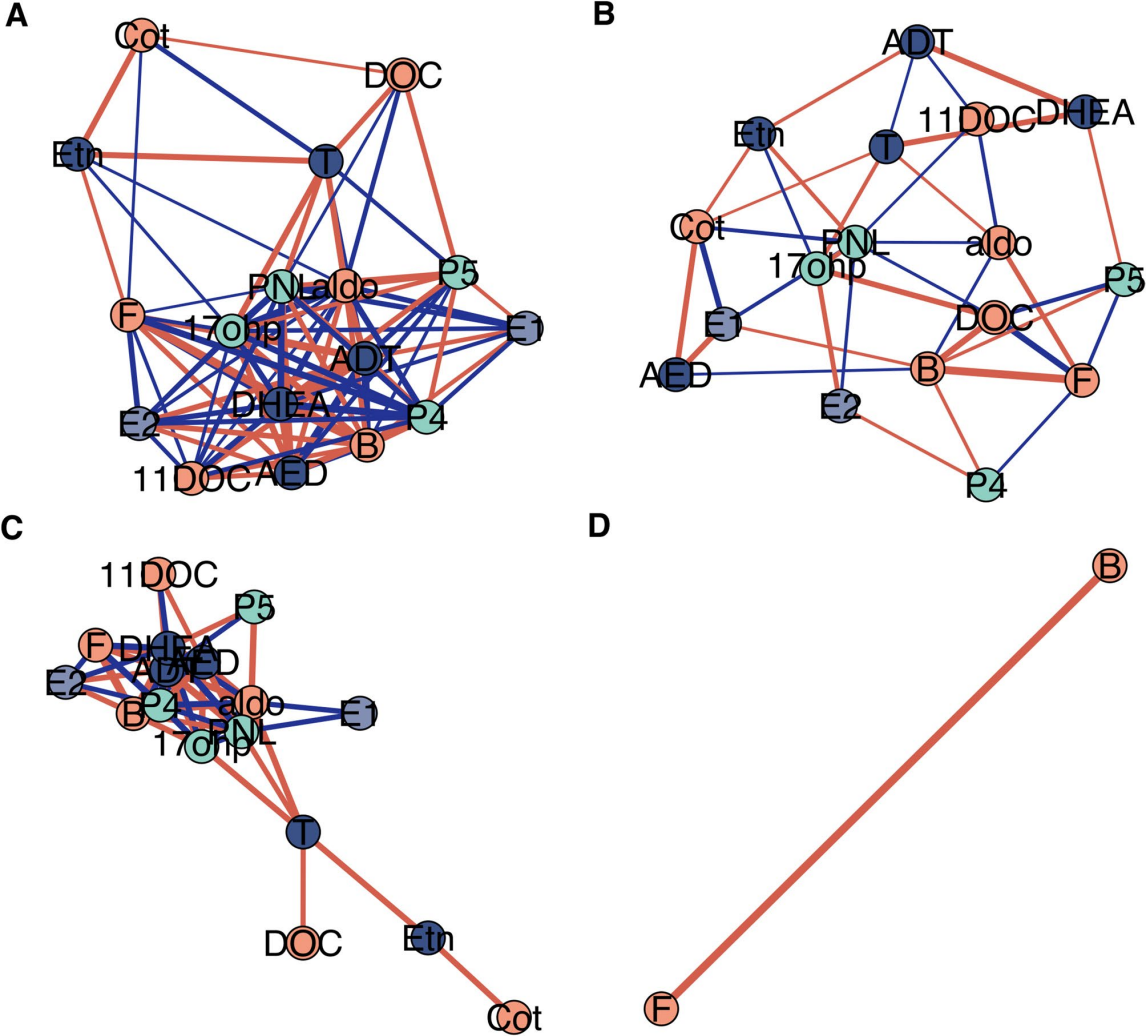
Steroid level comparisons in myalgic encephalomyelitis / chronic fatigue syndrome and control groups. (**A**) Absolute (untransformed) steroid levels in ME/CFS vs. control cohorts cortisol, cortisone, DHEA, estrone, and estradiol are plotted on a 0-150 ng/mol y-axis (SEM), while all other steroids use a 0–10 ng/mol scale (SEM) (**B**) and (**C**) Heatmaps displaying normalized (z-score) steroid levels in the ME/CFS and control group, respectively. Rows represent individual samples clustered using complete linkage, and columns represent specific steroids. Blue indicates lower-than-average levels, white represents the mean, and red indicates higher-than-average levels

No significant differences in absolute (untransformed) steroid levels were obtained when comparing the two groups (ME/CFS & CTRL) using Mann-Whitney U Tests (Table 2, Fig. 1)

**Table 2:** Comparison of absolute steroid levels between myalgic encephalomyelitis/ chronic fatigue syndrome and control groups

STEROID	[CTRL] (mean)	[ME/CFS] (mean)	[CTRL] (std)	[ME/CFS] (std)	*p* value	Cohen’s d
Aldosterone (Aldo)	0.357	0.296	0.300	0.209	0.516	-0.236
Androsterone (ADT)	5.957	9.495	4.719	11.691	0.483	0.397
Androstenedione (AED)	0.773	1.072	0.429	1.133	0.509	0.349
Cortisol (F)	138.064	135.157	62.304	61.887	0.718	-0.047
Cortisone (Cot)	26.504	25.189	8.562	11.179	0.415	-0.132
Corticosterone (B)	2.970	3.000	3.202	2.806	0.829	0.01
1-deoxycorticosterone (DOC)	0.324	0.252	0.475	0.386	0.818	-0.166
11-deoxycortisol (11DOC)	0.708	0.509	0.840	0.516	0.591	-0.285
Dehydroepiandrosterone (DHEA)	20.713	27.484	26.800	31.127	0.284	0.233
Etiocholanolone (Etn)	2.433	2.322	2.467	2.295	0.992	-0.047
17α-hydroxyprogesterone (17OHP)	0.381	0.389	0.207	0.361	0.332	0.027
Pregnanolone (PNL)	1.695	0.644	2.332	1.218	0.095	-0.565
Pregnenolone (P_5)_	4.131	4.152	8.919	5.265	0.327	0.003
Progesterone (P_4)_	0.477	1.473	0.193	3.295	0.642	0.427
Testosterone (T)	1.694	1.892	2.193	2.873	0.837	0.077
Estrone (E1)*	82.727	45.340	167.791	39.091	0.876	-0.307
Estradiol (E2)*	30.254	41.060	30.420	67.805	0.445	0.206

Abbreviations: Aldo; aldosterone, ADT; androsterone, AED; androstenedione, F; cortisol, Cot; cortisone, B; corticosterone, DOC; 1-deoxycorticosterone, 11DOC; 11-deoxycortisol, DHEA; dehydroepiandrosterone, Etn; etiocholanolone, 17OHP; 17α-hydroxyprogesterone, PNL; pregnanolone, P_5_; pregnenolone, P_4_; progesterone, T; testosterone, E1; estrone, E2; estradiol * ng/mol

### Gender stratified analysis of absolute steroid levels

Significant differences in absolute levels of four steroids were demonstrated between the female and male cohorts (ME/CFS and CTRL cohorts combined) (Table 3). Androsterone (Female: mean = 0.66ng/ml, Male: 1.00ng/ml, adjusted p value 0.012), DHEA (Female: mean = 0.99ng/ml, Male: 1.58ng/ml, adjusted p value < 0.0001), OHP (Female: mean = 0.11ng/ml, Male: 0.17ng/ml, adjusted p value = 0.04), and testosterone (Female: mean = 0.13ng/ml, Male: 0.72ng/ml, adjusted p value < 0.0001) were all demonstrated to have gender effect (Table 3.).

**Table 3:** Steroid level comparisons by reported gender (combined ME/CFS and CTRL) cohorts)

Steroid [ng/mol]	[Mean] (Female)	[Male] (Mean)	[Female] (STD)	[Male] (STD)	*p* value	q value
Aldosterone (Aldo)	0.320	0.338	0.192	0.364	0.505	0.734
Androsterone (ADT)	5.273	12.633	5.204	12.620	0.002	0.011
Androstenedione (AED)	0.984	0.800	1.040	0.260	0.654	0.805
Cortisol (F)	146.357	117.119	58.933	63.607	0.028	0.075
Cortisone (Cot)	25.875	25.790	9.972	9.993	0.818	0.889
Corticosterone (B)	3.155	2.648	2.671	3.587	0.129	0.294
11-Deoxycorticosterone (DOC)	0.619	0.589	0.728	0.649	0.913	0.913
Dehydroepiandrosterone (DHEA)	11.804	48.687	8.496	38.821	0.000	0.000
Etiocholanolone (Etn)	1.855	3.422	2.163	2.451	0.021	0.069
17α-Hydroxyprogesterone (17OHP)	0.320	0.513	0.276	0.285	0.010	0.038
Pregnanolone (PNL)	1.170	1.170	2.142	1.422	0.465	0.734
Pregnenolone (P5)	4.170	4.083	8.092	5.401	0.623	0.805
Progesterone (P4)	1.251	0.423	2.867	0.127	0.152	0.304
Testosterone (T)	0.350	4.678	0.209	2.580	0.000	0.000
Estrone (E1)*	75.323	41.453	148.100	22.024	0.833	0.889
Estradiol (E2)*	43.327	20.319	60.036	27.139	0.330	0.586

* pmol/ml

Adjusted *p* value; False Discovery Rate (FDR) = BH q < 0.05)

**Fig. 2:**
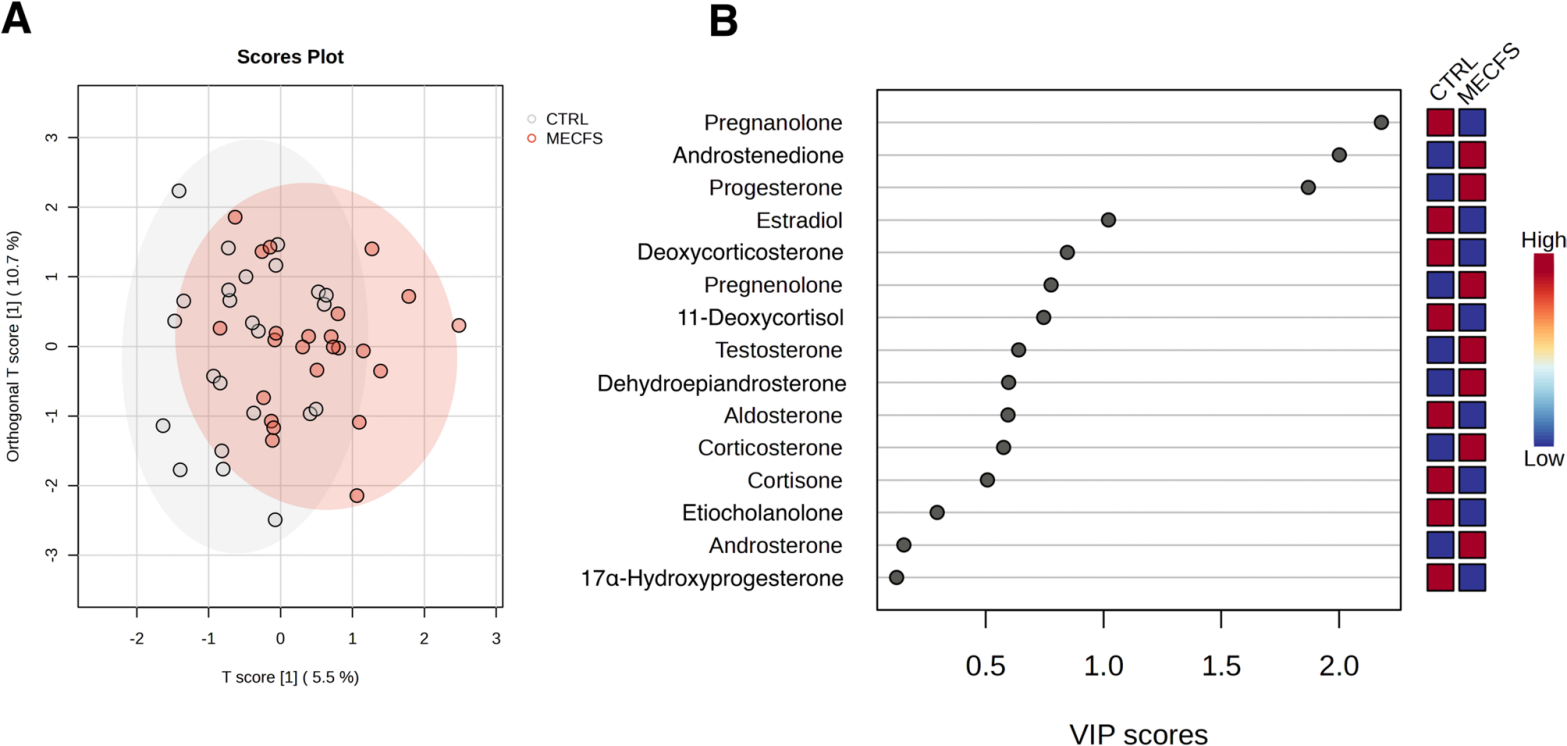
Absolute steroid level comparison in ME/CFS vs. control cohorts by gender. (**A**) Females and (**B**) Males. Steroid levels are reported in ng/mol, except for estradiol and estrone, which are shown in pmol/mol. Cortisol, cortisone, DHEA, estrone, and estradiol are plotted on a 0 -150 ng/mol y-axis, while all other steroids are displayed on a 0–10 ng/mol scale

Known differences in steroid levels and hormone dynamics exist between males and females, as well as across different ages and reproductive stages [35]. Significant differences in three steroid levels were observed between female and male participants in the combined ME/CFS and CTRL cohorts using Mann-Whitney U tests. Therefore, the data was stratified by gender, and separate analyses were conducted for the female and male cohorts. No significant differences in absolute (untransformed) steroid levels were obtained when comparing the two groups (ME/CFS & CTRL) using Mann-Whitney U Tests in the female cohort (Table 4) or male cohort (Table 5). (Figure 2) No correlates were found between age and steroid in this cohort, therefore no subgrouping based on age was conducted, nor was age controlled for (data not shown). 

**Table 4:** ME/CFS and control comparison in absolute steroid levels in females

Steroid	CTRL (mean)	ME/CFS (mean)	CTRL (STD)	ME/ CFS (STD)	*p* value	Cohen’s d
Aldosterone (Aldo)	0.316	0.324	0.175	0.212	0.940	0.041
Androsterone (ADT)	4.708	5.837	4.417	5.983	0.763	0.215
Androstenedione (AED)	0.750	1.218	0.491	1.369	0.291	0.455
Cortisol (F)	139.623	153.090	59.494	59.510	0.440	0.226
Cortisone (Cot)	26.581	25.168	8.450	11.536	0.407	-0.140
Corticosterone (B)	2.683	3.624	2.035	3.185	0.318	0.352
11-Deoxycorticosterone (DOC)	0.216	0.140	0.416	0.163	0.911	-0.241
11-Deoxycortisol (11DOC)	0.703	0.534	0.885	0.549	0.880	-0.229
Dehydroepiandrosterone (DHEA)	10.197	13.411	6.556	10.035	0.346	0.379
Etiocholanolone (Etn)	1.848	1.863	2.440	1.928	0.608	0.007
17α-Hydroxyprogesterone (17OHP)	0.296	0.345	0.186	0.348	0.692	0.176
Pregnanolone (PNL)	1.714	0.624	2.650	1.350	0.205	-0.518
Pregnenolone (P5)	4.136	4.205	10.392	5.227	0.297	0.008
Progesterone (P4)	0.508	1.994	0.211	3.971	0.692	0.528
Testosterone (T)	0.313	0.388	0.137	0.261	0.678	0.360
Estrone (E1)*	103.031	47.616	204.172	44.784	1.000	-0.375
Estradiol (E2)*	38.288	48.366	34.119	78.934	0.415	0.166

Abbreviations: ME/CFS; Myalgic Encephalomyelitis/ Chronic Fatigue Syndrome, STD; standard deviation, Steroid concentrations are reported in nmol/mL (pmol/mL for estrone* and estradiol*)

**Table 5:** Absolute steroid level comparison between myalgic encephalomyelitis/ chronic fatigue syndrome and control groups in males

Steroid	CTRL (mean)	ME/CFS (mean)	CTRL (STD)	ME/ CFS (STD)	*p* value	Cohen’s d
Aldosterone (Aldo)	0.440	0.240	0.467	0.202	0.227	-0.556
Androsterone (ADT)	8.454	16.810	4.549	16.753	0.189	0.681
Androstenedione (AED)	0.819	0.779	0.292	0.243	0.713	-0.149
Cortisol (F)	134.946	99.290	71.784	52.822	0.189	-0.566
Cortisone (Cot)	26.350	25.230	9.373	11.201	0.431	-0.108
Corticosterone (B)	3.544	1.753	4.926	1.207	0.564	-0.499
11-Deoxycorticosterone (DOC)	0.540	0.475	0.539	0.590	0.784	-0.115
11-Deoxycortisol (11DOC)	0.719	0.459	0.801	0.473	0.493	-0.395
Dehydroepiandrosterone (DHEA)	41.745	55.629	38.854	40.122	0.156	0.352
Etiocholanolone (Etn)	3.603	3.240	2.213	2.810	0.792	-0.144
17α-Hydroxyprogesterone (17OHP)	0.551	0.476	0.127	0.395	0.226	-0.256
Pregnanolone (PNL)	1.656	0.684	1.681	0.982	0.217	-0.706
Pregnenolone(P5)	4.123	4.045	5.474	5.703	0.956	-0.014
Progesterone (P4)	0.415	0.431	0.140	0.120	0.916	0.123
Testosterone (T)	4.455	4.901	1.640	3.385	0.958	0.168
Estrone (E1)	42.119	40.788	18.724	26.226	0.792	-0.058
Estradiol (E2)	14.188	26.450	10.175	37.266	0.916	0.449

Abbreviations: ME/CFS; Myalgic Encephalomyelitis/ Chronic Fatigue Syndrome, STD; standard deviation, Steroid concentrations are reported in nmol/mL (pmol/mL for estrone* and estradiol*)

### Comparison of steroid: steroid ratio levels between ME/CFS and control groups

The ratio of cortisol to pregnanolone (cortisol/pregnanolone) was significantly higher in the ME/CFS group (mean = 72.843) compared to the CTRL group (mean = 50.069). Additionally, the ratio of pregnanolone to progesterone (pregnanolone /progesterone) was significantly lower in the MECFS group (mean = 0.876) compared to the CTRL group (mean = 1.797) (p value of 0.036). After adjusting for multiple comparisons using the false discovery rate (FDR), these differences did not retain statistical significance (Table 6).

**Table 6:** Transformed steroid ratio comparisons between ME/CFS and control groups

Steroid Ratio	Control (Mean)	ME/CFS (Mean)	Control (STD)	ME/CFS (STD)	*p*-value
Cortisol / Pregnanolone	50.069	72.843	46.922	43.959	0.042
Pregnanolone / Progesterone	1.797	0.876	1.897	1.458	0.036

Abbreviations: ME/CFS, Myalgic Encephalomyelitis/Chronic Fatigue Syndrome; F, Cortisol; PNL, Pregnenolone; P4, Progesterone; STD, Standard Deviation

### Steroid ratio comparisons between ME/CFS and control group: female

Transformed mean values for steroid ratios in the female ME/CFS and control groups, and unadjusted *p*-values are outlined in Table 7. Steroid ratios are calculated as the concentration of one steroid relative to another.

**Table 7:** Steroid ratio comparisons between female ME/CFS and female control groups

Ratio	ME/CFS Ratio (Mean)	Control Ratio (Mean)	*p*-value
Cortisol / Pregnanolone	78.813	56.037	0.0387

Abbreviations: ME/CFS, Myalgic Encephalomyelitis/Chronic Fatigue Syndrome

### Steroid ratio comparisons between ME/CFS and control group: male

No significant differences (unadjusted nor adjusted) were demonstrated between the ME/CFS and control group in the male cohort.

### Partial spearman correlation analysis of steroid interrelationships and concentration network analysis

**Fig. 3:**
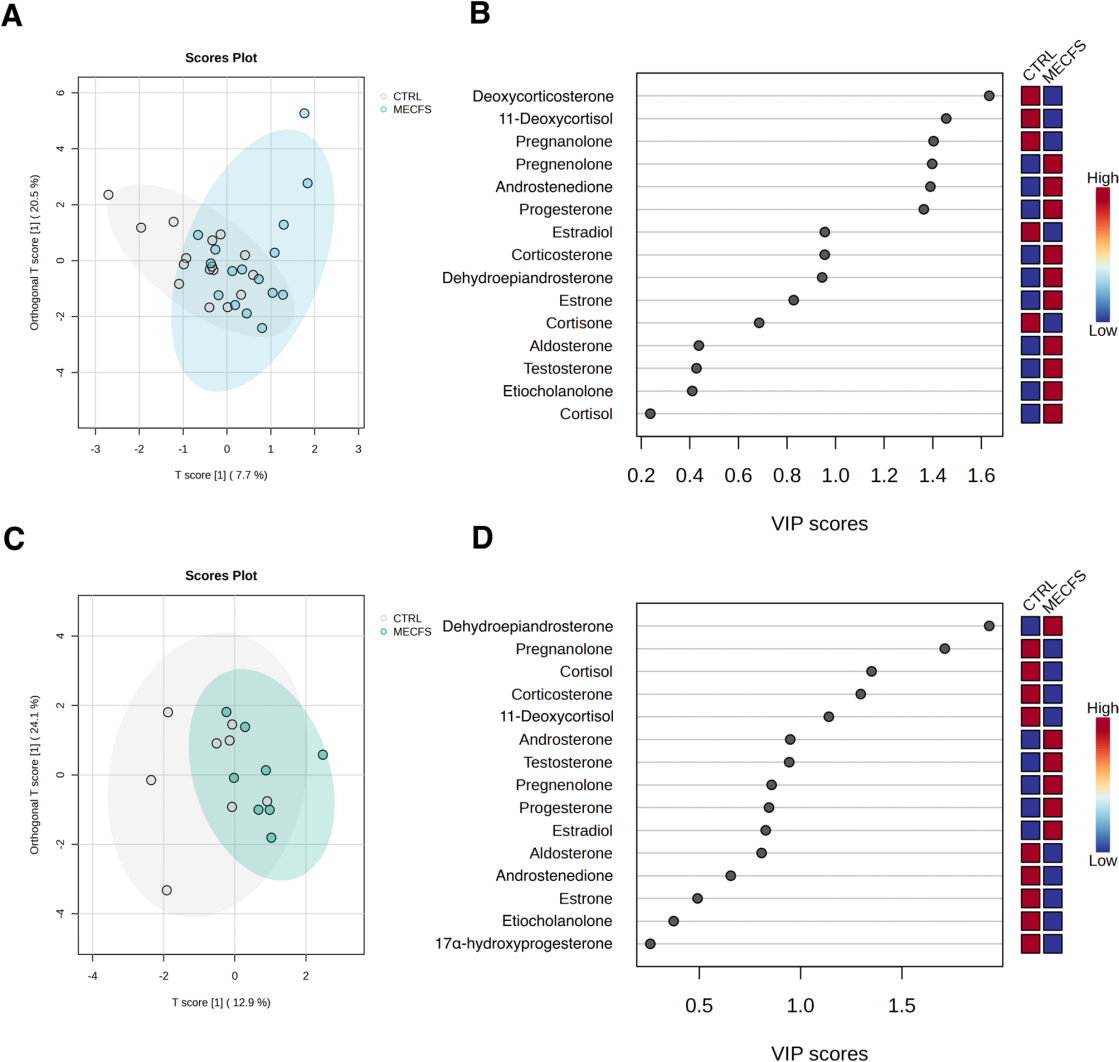
Steroid concentration network analysis based on spearman partial correlations. (**A**) (**B**) Network analysis for the control and ME/CFS groups, respectively, where nodes represent individual steroids and edges represent partial correlations with *r* > 0.3. (**C**) (**D**) Network analysis for the control and ME/CFS groups, respectively, where edges represent only significant correlations (*r* > 0.3, q < 0.3). These figures highlight the higher density and number of correlations in the control group compared to ME/CFS (**A** vs. **B**). Additionally, more significant correlations are observed in the control group (**C** vs. **D**), whereas the ME/CFS group only shows correlations between cortisol and corticosterone. Abbreviations: ME/CFS; myalgic encephalomyelitis/ chronic fatigue syndrome. Aldo; aldosterone, ADT; androsterone, AED; androstenedione, F; cortisol, Cot; cortisone, B; corticosterone, DOC; 1-deoxycorticosterone, 11DOC; 11-deoxycortisol, DHEA; dehydroepiandrosterone, Etn; etiocholanolone, 17OHP; 17α-hydroxyprogesterone, PNL; pregnanolone, P_5_; pregnenolone, P_4_; progesterone, T; testosterone, E1; estrone, E2; estradiol

52 significant partial correlations were demonstrated within the control group (Table 8), whilst only one partial correlation between cortisol and corticosterone was marginally significant in the ME/CFS cohort (*r* = 0.885, *p* = 0.002, q = 0.204) (Table 9). (Figure 3) There were 57 significant partial correlations that were demonstrated to be different between groups (ME/CFS vs. CTRL) (Table 10). 

**Table 8:** Significant partial correlations within the control group

Steroid 1	Steroid 2	*r*	*p* value	q value
Androstenedione (AED)	Dehydroepiandrosterone (DHEA)	0.960	0.000	0.006
Androstenedione (AED)	Androsterone (ADT)	-0.852	0.004	0.080
Androstenedione (AED)	Corticosterone (B)	-0.845	0.004	0.080
Androstenedione (AED)	Progesterone (P4)	0.854	0.003	0.080
Androsterone (ADT)	Dehydroepiandrosterone (DHEA)	0.850	0.004	0.080
Cortisol (F)	Corticosterone (B)	0.893	0.001	0.080
Corticosterone (B)	Progesterone (P4)	0.864	0.003	0.080
Aldosterone (Aldo)	17α-Hydroxyprogesterone (17OHP)	-0.830	0.006	0.096
Dehydroepiandrosterone (DHEA)	Progesterone (P4)	-0.821	0.007	0.101
Aldosterone (Aldo)	Pregnenolone (PLN)	-0.805	0.009	0.121
Androstenedione (AED)	Pregnenolone (PLN)	0.797	0.010	0.124
Androstenedione (AED)	17α-Hydroxyprogesterone (17OHP)	0.783	0.013	0.131
Dehydroepiandrosterone (DHEA)	Corticosterone (B)	0.787	0.012	0.131
17α-Hydroxyprogesterone (17OHP)	Pregnenolone (PLN)	-0.757	0.018	0.176
Androstenedione (AED)	Aldosterone (Aldo)	0.746	0.021	0.182
Cortisol (F)	Progesterone (P4)	-0.743	0.022	0.182
Dehydroepiandrosterone (DHEA)	Pregnenolone (PLN)	-0.740	0.023	0.182
Aldosterone (Aldo)	Pregnenolone (P5)	0.705	0.034	0.210
Androstenedione (AED)	Cortisol (F)	0.713	0.031	0.210
Androsterone (ADT)	17α-Hydroxyprogesterone (17OHP)	0.707	0.033	0.210
Androsterone (ADT)	Progesterone (P4)	0.712	0.032	0.210
Dehydroepiandrosterone (DHEA)	Cortisol (F)	-0.709	0.032	0.210
17α-Hydroxyprogesterone (17OHP)	Progesterone (P4)	-0.696	0.037	0.221
Aldosterone (Aldo)	Progesterone (P4)	-0.679	0.044	0.223
Corticosterone (B)	17α-Hydroxyprogesterone (17OHP)	0.684	0.042	0.223
Testosterone (T)	Aldosterone (Aldo)	0.687	0.041	0.223
Testosterone (T)	17α-Hydroxyprogesterone (17OHP)	0.681	0.043	0.223

Adjusted p value; FDR = BH q < 0.3)

**Table 9:** Significant partial correlations within the ME/CFS group

Steroid 1	Steroid 2	*r*	*p* value	q value
Corticosterone (B)	Cortisol (F)	0.885	0.002	0.204

Adjusted p value; False Discovery Rate (FDR) = BH q < 0.05)

**Table 10:** Significant differences in partial correlations between steroid pairs in myalgic Encephalomyelitis / Chronic fatigue syndrome and control groups

Steroid 1	Steroid 2	*r* ME/CFS	*r* CTRL	Z statistic	*p* value	CI Lower	CI Upper	q value
17α-Hydroxyprogesterone (17OHP)	Pregnenolone (PNL)	0.534	-0.757	5.136	0.000	0.848	1.562	0.000
Androstenedione (Andro)	Androstenedione (AED)	0.168	-0.852	4.643	0.000	0.567	1.396	0.000
Androstenedione (Andro)	Progesterone (P4)	-0.102	0.854	-4.455	0.000	-1.349	-0.509	0.000
Aldosterone (Aldo)	17α-Hydroxyprogesterone (17OHP)	0.091	-0.830	4.145	0.000	0.465	1.318	0.001
Androstenedione (AED)	Dehydroepiandrosterone (DHEA)	-0.013	0.850	-4.118	0.000	-1.273	-0.424	0.001
Dehydroepiandrosterone (DHEA)	Progesterone (P4)	0.071	-0.821	3.990	0.000	0.434	1.294	0.001
Androsterone (ADT)	Dehydroepiandrosterone (DHEA)	0.645	0.960	-3.817	0.000	-0.635	-0.121	0.003
Androsterone (ADT)	Pregnenolone (PNL)	-0.051	0.797	-3.703	0.000	-1.258	-0.384	0.004
Aldosterone (Aldo)	Progesterone (P4)	0.287	-0.679	3.637	0.000	0.451	1.339	0.004
11-Deoxycorticosterone (DOC)	Pregnenolone (P5)	-0.469	0.548	-3.642	0.000	-1.368	-0.486	0.004
Androsterone (ADT)	Aldosterone (Aldo)	-0.111	0.746	-3.481	0.001	-1.262	-0.367	0.006
Corticosterone (B)	17α-Hydroxyprogesterone (17OHP)	-0.231	0.684	-3.475	0.001	-1.303	-0.400	0.006
Dehydroepiandrosterone (DHEA)	Corticosterone (B)	-0.002	0.787	-3.456	0.001	-1.209	-0.327	0.006
Androsterone (ADT)	Corticosterone (B)	-0.178	-0.845	3.432	0.001	0.263	1.097	0.006
Estradiol (E2)	17α-Hydroxyprogesterone (17OHP)	0.533	-0.427	3.405	0.001	0.418	1.329	0.006
Aldosterone (Aldo)	Corticosterone (B)	-0.342	0.586	-3.334	0.001	-1.308	-0.389	0.006
Androsterone (ADT)	11-Deoxycortisol (11DOC)	-0.315	0.610	-3.351	0.001	-1.306	-0.389	0.006
Cortisol (F)	11-Deoxycorticosterone (DOC)	-0.691	0.182	-3.351	0.001	-1.272	-0.361	0.006
Estradiol (E2)	Progesterone (P4)	0.372	-0.565	3.338	0.001	0.394	1.313	0.006
11-Deoxycorticosterone (DOC)	17α-Hydroxyprogesterone (17OHP)	0.634	-0.254	3.265	0.001	0.357	1.281	0.007
Dehydroepiandrosterone (DHEA)	Cortisol (F)	0.073	-0.709	3.107	0.002	0.283	1.202	0.012
Androsterone (ADT)	17α-Hydroxyprogesterone (17OHP)	0.134	0.783	-2.977	0.003	-1.085	-0.210	0.018
Aldosterone (Aldo)	Cortisol (F)	0.538	-0.293	2.923	0.003	0.274	1.236	0.018
Androstenedione (AED)	Estradiol (E2)	-0.287	0.543	-2.926	0.003	-1.235	-0.275	0.018
Corticosterone (B)	Progesterone (P4)	0.384	0.864	-2.935	0.003	-0.894	-0.144	0.018
Testosterone (T)	Cortisone (Cot)	0.309	-0.531	2.952	0.003	0.284	1.243	0.018
Testosterone (T)	11-Deoxycorticosterone (DOC)	-0.239	0.570	-2.891	0.004	-1.221	-0.260	0.019
17α-Hydroxyprogesterone (17OHP)	Progesterone (P4)	-0.025	-0.696	2.704	0.007	0.178	1.109	0.033
Androstenedione (AED)	17α-Hydroxyprogesterone (17OHP)	0.061	0.707	-2.654	0.008	-1.086	-0.162	0.037
Corticosterone (B)	11-Deoxycorticosterone (DOC)	0.694	0.042	2.639	0.008	0.161	1.093	0.038
Dehydroepiandrosterone (DHEA)	11-Deoxycortisol (11DOC)	0.070	-0.629	2.624	0.009	0.172	1.135	0.038
Corticosterone (B)	Pregnenolone (P5)	0.382	-0.377	2.591	0.010	0.183	1.183	0.041
Aldosterone (Aldo)	11-Deoxycorticosterone (DOC)	0.213	-0.521	2.574	0.010	0.172	1.164	0.041
Aldosterone (Aldo)	Pregnenolone (PNL)	-0.324	-0.805	2.516	0.012	0.099	0.910	0.045
Corticosterone (B)	Estradiol (E2)	-0.039	0.626	-2.508	0.012	-1.107	-0.141	0.045
Dehydroepiandrosterone (DHEA)	Pregnenolone (PNL)	-0.175	-0.740	2.503	0.012	0.116	1.008	0.045
Etiocholanolone (Etn)	Pregnenolone (PNL)	0.447	-0.289	2.523	0.012	0.161	1.166	0.045
Cortisol (F)	Estradiol (E2)	0.032	-0.616	2.434	0.015	0.122	1.094	0.053
Pregnenolone (PNL)	Progesterone (P4)	0.037	-0.612	2.430	0.015	0.121	1.095	0.053
Cortisol (F)	17α-Hydroxyprogesterone (17OHP)	0.245	-0.429	2.296	0.022	0.095	1.119	0.074
11-Deoxycortisol (11DOC)	17α-Hydroxyprogesterone (17OHP)	0.292	-0.376	2.253	0.024	0.083	1.114	0.081
Etiocholanolone (Etn)	Testosterone (T)	0.116	0.665	-2.219	0.026	-1.002	-0.060	0.086
Androsterone (ADT)	Cortisol (F)	0.219	0.713	-2.173	0.030	-0.942	-0.044	0.090
Androsterone (ADT)	Pregnenolone (P5)	0.025	-0.569	2.175	0.030	0.055	1.051	0.090
Corticosterone (B)	11-Deoxycortisol (11DOC)	-0.107	0.514	-2.187	0.029	-1.075	-0.060	0.090
Aldosterone (Aldo)	Pregnenolone (P5)	0.216	0.705	-2.128	0.033	-0.938	-0.035	0.094
Androstenedione (AED)	Cortisone (Cot)	0.624	0.074	2.129	0.033	0.040	1.008	0.094
Dehydroepiandrosterone (DHEA)	Estradiol (E2)	-0.038	-0.603	2.140	0.032	0.043	1.024	0.094
Androstenedione (AED)	Progesterone (P4)	0.237	0.712	-2.104	0.035	-0.924	-0.029	0.098
Etiocholanolone (Etn)	Cortisol (F)	-0.197	0.414	-2.074	0.038	-1.070	-0.029	0.104
Androstenedione (AED)	Pregnenolone (P5)	0.081	-0.505	2.062	0.039	0.025	1.047	0.105
Cortisone (Cot)	Estrone (E1)	-0.623	-0.098	-2.044	0.041	-0.985	-0.018	0.107
Androstenedione (AED)	Pregnenolone (PNL)	0.183	0.667	-2.011	0.044	-0.941	-0.009	0.114
Cortisol (F)	11-Deoxycortisol (11DOC)	0.190	-0.395	1.976	0.048	0.000	1.050	0.121
Cortisol (F)	Pregnenolone (P5)	-0.395	0.185	-1.957	0.050	-1.046	0.005	0.121
Cortisol (F)	Progesterone (P4)	-0.340	-0.743	1.953	0.051	-0.003	0.839	0.121
Pregnenolone (P5)	Progesterone (P4)	-0.003	0.539	-1.963	0.050	-1.009	0.003	0.121

Adjusted p value; FDR = BH q < 0.05

### Network centrality measures of ME/CFS and control groups

Centrality metrics, including degree, betweenness, closeness, and eigenvector centrality, were calculated separately for the ME/CFS and control groups based on partial Spearman correlation networks (*r* > 0.3). The resulting measures (Table 11) highlighted the relative central roles and connectivity of each steroid within the network for each group.

**Table 11:** Full network centrality measures of ME/CFS and control groups

Steroid	Degree (CTRL)	Degree (ME/CFS)	Betweeness (CTRL)	Betweeness (ME/CFS)	Closeness (CTRL)	Closeness (ME/CFS)	Eigen (CTRL)	Eigen (ME/CFS)
ADT	13	4	2.470	4.250	0.053	0.028	0.975	0.384
AED	11	3	0.493	3.900	0.048	0.029	0.873	0.306
Etn	5	5	0.967	11.150	0.037	0.036	0.304	0.566
DHEA	12	5	1.070	10.633	0.050	0.037	0.931	0.727
T	9	3	5.250	1.450	0.043	0.029	0.549	0.434
F	11	4	9.667	4.300	0.048	0.031	0.768	0.559
aldo	14	5	8.444	4.300	0.056	0.033	0.962	0.678
Cot	4	7	1.033	16.733	0.036	0.037	0.170	0.855
B	12	5	1.070	8.933	0.050	0.033	0.931	0.651
11DOC	10	5	0.100	8.067	0.045	0.036	0.808	0.772
E1	9	3	0.500	4.400	0.043	0.030	0.712	0.429
E2	10	3	0.100	2.833	0.045	0.031	0.808	0.434
DOC	5	4	1.167	3.317	0.037	0.033	0.307	0.605
17ohp	14	5	5.637	8.350	0.056	0.036	1.000	0.684
PNL	14	8	6.054	23.317	0.056	0.040	1.000	1.000
P5	11	4	2.908	6.233	0.048	0.031	0.807	0.537
P4	12	3	1.070	2.833	0.050	0.028	0.931	0.404

Abbreviations: ME/CFS; myalgic encephalomyelitis/ chronic fatigue syndrome. Aldo; aldosterone, ADT; androsterone, AED; androstenedione, F; cortisol, Cot; cortisone, B; corticosterone, DOC; 1-deoxycorticosterone, 11DOC; 11-deoxycortisol, DHEA; dehydroepiandrosterone, Etn; etiocholanolone, 17OHP; 17α-hydroxyprogesterone, PNL; pregnanolone, P_5_; pregnenolone, P_4_; progesterone, T; testosterone, E1; estrone, E2; estradiol

### Orthogonal partial least squares discriminant analysis

**Fig. 4:**
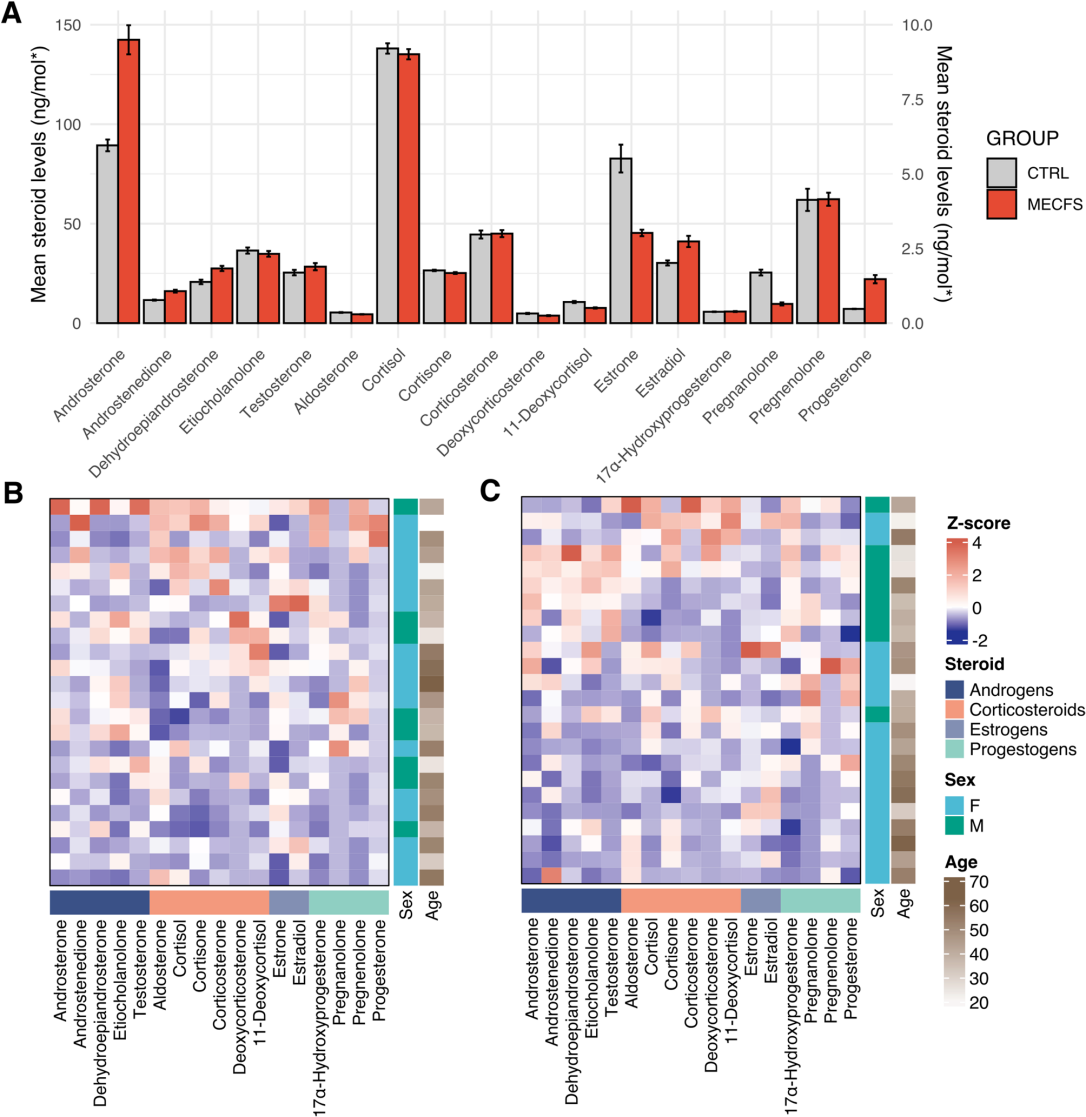
(**A**) Scores plot from the orthogonal partial least squares discriminant analysis (OPLS-DA) showing group separation between ME/CFS and control. (**B**) VIP scores plot from OPLS-DA identifying the most important variables driving group separation. Variables with VIP > 1.2 are considered significant contributors to the model

The Orthogonal Partial Least Squares Discriminant Analysis (OPLS-DA) model for the combined cohort (male and female) of control and ME/CFS groups demonstrated 5.49% of the variance in the predictor variables (steroids) (R²X = 0.0549) and 22.8% of the variance in the response variable (R²Y = 0.228) (primary component), indicating that 22.8% of the variance in group classification is explained by steroid levels (Fig. 4). Predictive power was poor, with a negative Q² value (Q² = -0.268). The orthogonal component explained 10.7% of the variance in the predictor variables (R²X[ortho] = 0.107) and 8.34% of the variance in the response variable (R²Y[ortho] = 0.0834), with a marginally positive predictive power (Q²[ortho] = 0.01451). When feature selection was adopted using > 0.3 Cohen’s d (steroids androsterone, androstenedione, pregnanolone, progesterone, estrone) model interpretability of X and Y variable datasets was R²X = 0.22, R²Y = 0.132, respectively, and the model predictability was Q² = 0.0362.

Variables with VIP scores greater than 1.2 are considered key contributors to the separation between ME/CFS and CTRL groups, indicating their importance in distinguishing between the two conditions. VIP > 1.2 are outlined in Table 12.

**Table 12:** OPLS-DA VIP threshold VIP[t] = > 1.2

Steroid	V1	V2
Pregnenolone	2.179	0.360
Androstenedione	2.001	0.104
Progesterone	1.869	0.480

### Orthogonal partial least squares discriminant analysis: female cohort and male cohort stratified

**Fig. 5:**
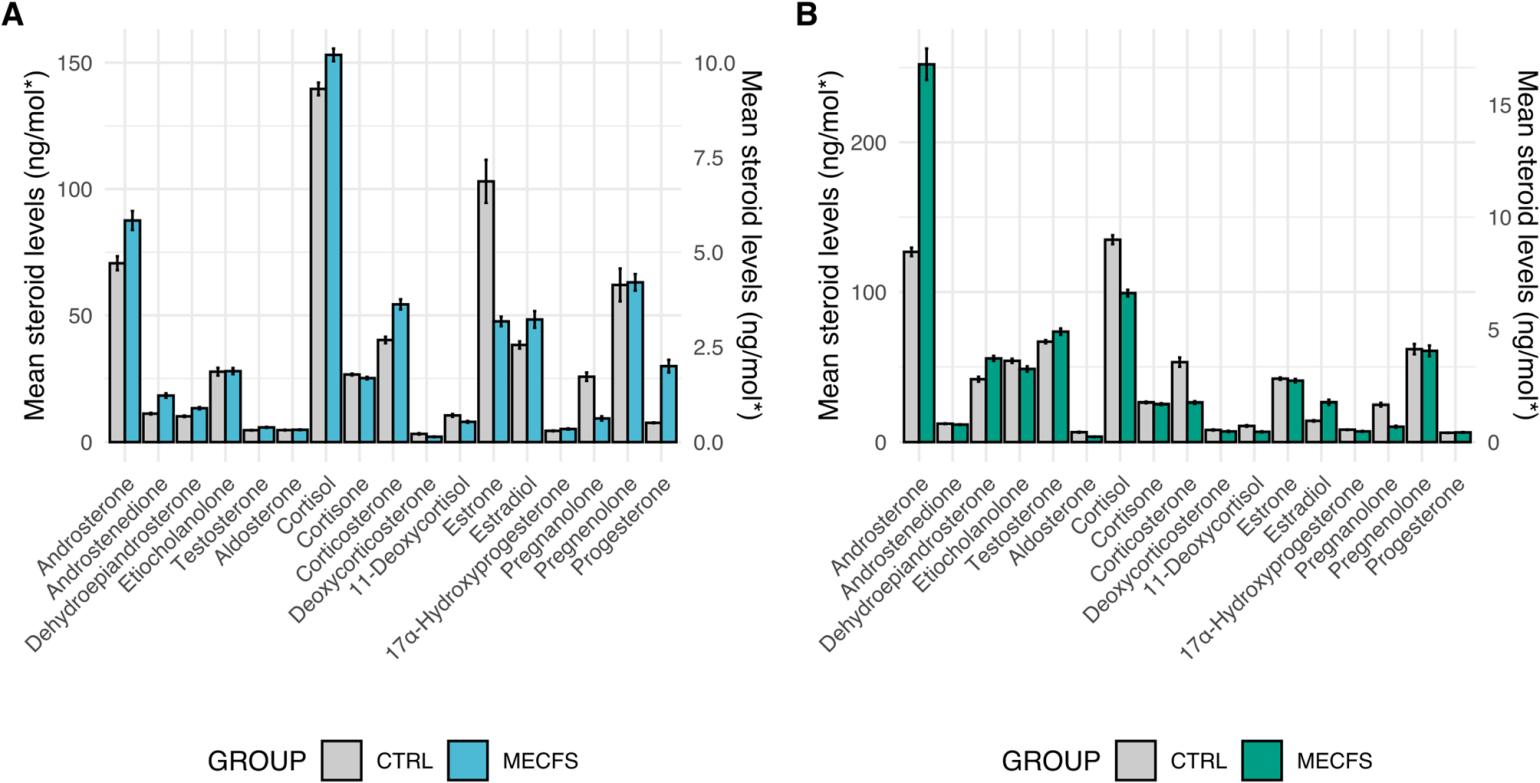
OPLS-DA Scores and VIP plots for ME/CFS vs. control groups by gender. (**A**) Scores plot (Females) from orthogonal partial least squares discriminant analysis (OPLS-DA) showing group separation between ME/CFS and Control. (**B**) VIP Scores plot (Females) from OPLS-DA highlighting the most important variables contributing to group separation. (**C**) Scores plot (Males) from OPLS-DA showing group separation between ME/CFS and Control. (**D**) VIP scores plot (Males) from OPLS-DA highlighting the most important variables contributing to group separation

The OPLS-DA model for the female cohort showed that the primary component explained 7.7% of the variance in the predictor variables (R²X = 0.077) and 22.8% of the variance in the response variable (R²Y = 0.228). However, the model’s predictive power was poor, with a negative Q² value (Q² = -0.313). The orthogonal component explained 20.5% of the variance in the predictor variables (R²X[ortho] = 0.205) and 13.0% of the variance in the response variable (R²Y[ortho] = 0.130), also with poor predictive power (Q²[ortho] = -0.364), indicating significant overfitting. (Fig. 5A and B).

For the male cohort, the OPLS-DA model’s primary component explained 12.9% of the variance in the predictor variables (R²X = 0.129) and 21.2% of the variance in the response variable (R²Y = 0.212). Predictive power was poor, with a negative Q² value (Q² = -0.562). The orthogonal component explained 24.1% of the variance in the predictor variables (R²X[ortho] = 0.241) and 18.0% of the variance in the response variable (R²Y[ortho] = 0.180), with a poor Q²[ortho] value (Q²[ortho] = -1.22). (Fig. 5C and D).

Variables with VIP scores greater than 1.2 are considered key contributors to the separation between ME/CFS and CTRL groups, indicating their importance in distinguishing between the two conditions. VIP Scores greater than 1.2 forfemale patients Vs controls are outlined in Table 13, and in Table 14 for male comparison.

**Table 13:** Female OPLS-DA VIP threshold VIP[t] = > 1.2

Steroid	V1	V2
11-Deoxycorticosterone (DOC)	1.633	0.815
11-Deoxycortisol (11DOC)	1.456	0.957
Pregnanolone (PNL)	1.404	0.232
Pregnenolone (P5)	1.398	0.933
Androstenedione (AED)	1.391	1.567
Progesterone (P4)	1.363	1.135

**Table 14:** Male OPLS-DA VIP threshold VIP[t] = > 1.2

Steroid	V1	V2
Dehydroepiandrosterone (DHEA)	1.931	0.191
Pregnanolone (PNL)	1.712	0.523
Cortisol (F)	1.351	1.417
Corticosterone (B)	1.297	1.693

## Discussion

### Absence of absolute steroid level differences in two-group comparison

No significant differences in absolute steroid levels were observed between the ME/CFS and control groups, either in the combined cohort or within gender-stratified groups. However, this may reflect limited statistical power rather than the absence of true biological differences.

To explore this, we estimated the sample sizes required to detect the effects sizes (Cohen’s d) derived from the present dataset (Table 2) using a significance threshold of α = 0.05 and a statistical power of 0.80. In the combined cohort, pregnanolone (d = 0.57), progesterone (d = 0.43), androsterone (d = 0.40), and androstenedione (d = 0.35) would require between 50 and 130 participants per group to reach statistical significance (Table 2. supplementary material). In the female cohort progesterone would require approximately 57 participants per group (d = 0.528), whilst pregnanolone would require an estimated 60 participants (d = -0.52) (Table 3. Supplementary material). In males, pregnanolone again showed a moderate to large effect size (d = -0.71) requiring an estimated 33 participants per group, whilst androsterone (d = 0.68, *n* ≈ 35), aldosterone (d = − 0.56, *n* ≈ 52), and cortisol (d = − 0.57, *n* ≈ 50) also all demonstrated moderate to large effects sizes (Table 4. Supplementary material). These results suggest that pregnanolone is consistently altered across sexes, whilst other steroids show sex-specific patterns.

### Steroid levels and clinical symptom severity

To explore potential associations between circulating steroid levels and symptom severity, we conducted exploratory spearman correlation analyses with clinical scores from the Mental Fatigue Scale and the FibroFatigue Scale. None of the associations remained significant after correction for multiple comparisons, and these results should be interpreted with considerable caution due to the small sample sizes. In the male ME/CFS subgroup (*n* = 6), some positive correlations were observed between fatigue scores and select corticosteroids, including cortisone, corticosterone, and 11-deoxycorticosterone (Table  Supplementary material 1A.- 1 C). Although these were not maintained after correction and therefore do not support strong or direct links between circulating steroid levels and fatigue severity, it does prompt further investigation in larger, symptom-stratified cohorts.

The only published steroidomics study in ME/CFS to date was recently conducted using gold-standard mass spectrometry (although employing liquid chromatography as opposed to supercritical fluid chromatography) [36] found group differences only when ME/CFS was stratified by gender *and* clinical severity (severe vs. mild/moderate ME/CFS). In this study, severe ME/CFS females exhibited higher levels of 11-deoxycortisol (compared to mild/moderate female ME/CFS and controls) and elevated 17-hydroxyprogesterone (compared to female controls) and mild/moderate ME/CFS females yielded higher progesterone levels compared to female controls. Males with mild/moderate ME/CFS had lower cortisol and corticosterone levels and higher progesterone compared to male controls. However, the study did not outline the criteria for defining clinical severity ME/CFS [36], limiting the interpretability of the findings.

Although the previous steroidomics study employed a different mass spectrometry platform and a distinct steroid panel, several of our findings are directionally consistent with theirs. In their study, lower cortisol levels were only observed in males with ME/CFS, which mirrors our finding of a larger Cohen’s *d* for cortisol in the male cohort. Progesterone was significantly elevated in both sexes in their analysis, whereas in our study, a moderate positive effect size for progesterone was observed only in females. Corticosterone also followed a similar pattern with their study reporting significantly lower levels in males, while our study showed moderate effect sizes in both sexes, with a stronger effect in males and a larger estimated sample size required to detect significance in females. In contrast, we did not replicate their findings for 17-hydroxyprogesterone or 11-deoxycortisol. Notably, their study did not include pregnanolone, which emerged as a key differentiator in our analysis. Finally, while their results were stratified by clinical severity, we were unable to perform such subgrouping in our study, which limits direct comparison. Stratification by severity and duration on illness may have revealed more nuanced patterns and is recommended in future studies.

Research on cortisol, one of the most studied steroid hormones and potential biomarkers in ME/CFS, generally supports the presentation of mild hypocortisolism in patients [37] which is in contrast with the current findings. However, the evidence remains mixed and somewhat controversial, with studies showing variability in absolute cortisol levels, diurnal patterns, and responses, leading to ongoing debate about its role in the condition [38]. Many of these studies [8, 39–42] used low specificity immunoassays that may have detected other glucocorticoid metabolites, leading to less accurate measurements. Immunoassays for cortisol often suffer from cross-reactivity with several corticosteroid metabolites, such as cortisone, 11-deoxycortisol, and corticosterone [18, 43]. This underscores the importance of using high specificity methods like mass spectrometry for accurate hormone profiling [44] which can elucidate the metabolism of HPA steroids. Further, the recent steroidomics study above [36] found that only *males* in the mild/moderate clinical subgroup had lowered cortisol (and corticosterone levels) compared to controls in severe ME/CFS participants, aligning with the present study. Majority of published studies on cortisol on ME/CFS, even if gender matched, did not stratify their analysis. Reanalysis of these results pertaining to gender specific effects are warranted. This highlights the importance of considering gender and sex in future study design and statistical analysis.

### Steroid ratio comparisons

Steroid hormone ratios can offer valuable insights into hormone network activity by reflecting relative production rates within the endocrine system [28]. In the combined cohort (females and males), ME/CFS patients compared to controls demonstrated a higher cortisol - to - pregnenolone (cortisol / pregnanolone) ratio or, conversely, a lower pregnanolone - to -cortisol (pregnanolone / cortisol) ratio. This difference appears to be driven by lower pregnanolone levels in ME/CFS (mean = 0.143 ng/ml) compared to controls (mean = 0.303 ng/ml), with cortisol levels remaining largely unchanged (ME/CFS mean = 2.09 ng/ml, CTRL mean = 2.10 ng/ml). Similarly, ME/CFS patients show a higher pregnanolone - to -progesterone (pregnanolone / progesterone) ratio or a lower progesterone -to - pregnanolone (progesterone /pregnanolone) ratio, driven by changes in both steroids (pregnanolone: ME/CFS mean = 0.143 ng/ml, CTRL mean = 0.303 ng/ml, prog: ME/CFS mean = 0.251 ng/ml, CTRL mean = 0.166 ng/ml). Whilst these findings suggest altered steroid metabolism in ME/CFS, they require validation in a larger sample size, as the observed differences did not remain significant after FDR correction. Increased statistical power and sample size are essential to confirm these preliminary findings, particularly if they are to be considered for diagnostic or biomarker development.

### Network dynamics

Based on partial correlations, the overall network patterns suggest distinct differences between the ME/CFS and control cohorts. While 52 significant direct steroid relationships were demonstrated within the control group, only one relationship between cortisol and corticosterone remained significant within the ME/CFS cohort (*r* = 0.885, *p*-value = 0.002, q = 0.204). 57 *between-group* steroid relationships were observed to be significantly different. This highlights extensive and robust relationships between steroids in controls, whist such relationships appear diminished or lost in the ME/CFS cohort. The exception is the cortisol-corticosterone relationship, where the strength and direction remain consistent across both groups.

From a statistical perspective, this loss of significant relationships in ME/CFS may be due to increased variability in steroid levels within the ME/CFS group, reducing the precision of correlation estimates and making it more difficult to achieve significance with FDR correction. Sample heterogeneity which may result from clinical severity or medications, can introduce additional variability, masking significant relationships that may be present within a more homogeneous group (e.g., a control group). At the biological level, significant between-group differences indicate distinct patterns of steroid interactions, suggesting changes in the biological processes underlying steroid regulation and metabolism. These results may reflect a loss of homeostasis in ME/CFS. Likely, the results reflect a combination of increased variability, and genuine biological differences in steroid regulation and metabolism. Despite these disruptions, the persistent cortisol-corticosterone relationship indicates some consistent aspects of steroid regulation.

There is growing evidence of broad biological abnormalities in ME/CFS patients, including immune, metabolism, and neuroendocrine disturbances, and these may lead to a more complex network of interactions among steroids compared to healthy controls [45]. In ME/CFS, regulatory disruptions such as chronic inflammation, viral persistence, or HPA axis dysfunction, may introduce additional feedback loops, further complicating steroid interactions [46]. When multiple interdependencies exist, as seen in the steroid network, the direct effect of one steroid on another can be masked by the influence of other steroids. While classical pair-wise spearman correlations capture both direct and indirect effects, partial correlations aim to isolate only direct effects. However, if the direct effect is small relative to the combined direct and indirect effects, it may become non-significant after ‘partial-ing out’ all other effects. This is potentially what is being observed in the patient cohort of the current dataset.

In healthy physiology, steroid hormones form an interdependent network, where changes in one hormone are typically met with compensatory shifts in others. This dynamic coordination, mediated by feedback loops, enzyme driven conversions, receptor density, and circadian timing [47], allows the endocrine system to maintain internal stability across a range of physiological demands. Partial correlation networks may capture this underlying coherence, highlighting how hormones change together rather than in isolation. In ME/CFS, however, the striking loss of these correlations suggests that this adaptive communication has broken down. This network collapse may be driven by biological disturbances observed in ME/CFS, including chronic inflammation [48] (which can regulate steroid hormone metabolism and action) [49], HPA axis dysfunction (disrupting central hormonal rhythms and the stress response), mitochondrial inefficiency [50] (hormone biosynthesis initially takes place in the inner membrane of the mitochondria), and β2-adrenergic receptor autoimmunity [51] (which may directly affect downstream glucocorticoid functioning) [52]. If the endocrine network becomes incoherent due to any of these disruptions it no longer is able to buffer, compensate, or recalibrate in response to stress or internal change. This loss of coordinated endocrine flexibility may underlie key clinical features of ME/CFS, which is supported by the fact that ME/CFS symptoms including impaired stress tolerance, sleep-wake disturbances, cognitive fog, and immune dysregulation overlap with symptoms seen in individuals with endocrine issues [6, 53, 54].

Centrality measures revealed differences in the coordination of steroid networks between ME/CFS and control groups. The ME/CFS network exhibited reduced centrality across multiple metrics, indicating reduced node (steroid) influence and overall network interactions relative to the control group. Of note, cortisone (degree centrality = 7 in ME/CFS vs. 4 in CTRL; betweenness = 16.7 vs. 1.03) emerged as highly central in ME/CFS, despite being largely peripheral in controls. Cortisone’s increased network centrality in ME/CFS, despite unchanged concentrations, suggests it may be playing a regulatory or compensatory role within a destabilised steroid network. This shift may reflect altered 11β-HSD enzyme activity, with impaired cortisol availability and disrupted interconversion between cortisol and cortisone. Future studies could assess this hypothesis by measuring 11β-HSD1 and 11β-HSD2 activity in ME/CFS. Progesterone (degree centrality = 3 in ME/CFS vs. 12 in CTRL; eigenvector centrality = 0.40 vs. 0.93) showed the opposite pattern, indicating reduced network integration in ME/CFS and suggesting possible dysregulation of the progestogen pathway. Collectively the results suggest dysregulation of HPA axis function and progestogen signalling, as reflected in altered partial correlations, centrality profiles, and steroid ratios.

Accounting for both gender/ sex specific hormone regulation and network dynamics is critical in ME/CFS research, given the well documented differences in hormone profiles. In our dataset, androsterone, DHEA, 17-OHP, and testosterone demonstrate significant gender differences, prompting us to analyse the cohort separately by self-reported gender. However, attempts to analyse partial correlations within and between ME/CFS and control groups for segregated female and male groups prevented reliable analysis of correlation estimates due to the high-dimensional nature of the data (the number of steroid variables exceeded the number of participants in each group which violates the assumptions of analysis leading to unstable estimates and spurious relationships). Future studies should account for sex and gender differences, conduct stratified analyses, and ensure sufficient sample sizes to support these approaches.

Given the well-established role of sex hormones in regulating immune, neuroendocrine, and metabolic systems, the strong female predominance in ME/CFS, and emerging evidence that menstrual cycle and reproductive phase influence symptom severity [6], accounting for these dynamics is essential to understanding sex-specific disease mechanisms. A more detailed exploration of sex-specific steroid dynamics, particularly at the network level, was beyond the scope of this sample size and study design but remains a key priority for future research. There is also a clear need for data on sex-specific steroid network dynamics in healthy individuals to support meaningful comparisons with pathological states, but which remains lacking. The authors are currently working toward this aim in cohorts designed to account for sex, reproductive status and menstrual cycle phase.

### OPLS-DA

The OPLS-DA model for the combined (female and male) cohort revealed a modest relationship between steroid levels and group classification. However, the model’s predictive power was poor, with a negative Q² value (Q² = -0.268), indicating overfitting and low generalisability. When feature selection was employed to reduce dimensionality and overfitting, the model demonstrated only marginal improvement Q² = 0.0362. For the split female and male datasets, the OPLS-DA model indicated a moderate relationship between steroid levels and group classification, although again, the model’s predictive power was very poor, with a negative Q² value (female; Q² = -0.313, male; Q² = -0.562). An R²Y value around 20% can be considered meaningful given the biological complexity of steroid networks and suggests that the models are identifying some underlying patterns, though a significant portion of variability remains unexplained. Splitting the data by gender further reduced our sample sizes. While the combined cohort shows some improvements in handling overfitting, the female only cohort demonstrates a stronger relationship between steroid levels and group classification. However, all models yielded poor predictive power overall, highlighting the need for further refinement of these OPLS-DA models. Despite being exploratory and limited in size, this dataset provides valuable insights due to the inherent biological complexity and variability of steroid hormone dynamics and merits further investigation.

### Pregnanolone

Pregnanolone emerged as a key distinguishing steroid in the ME/CFS cohort, showing differences at both univariate and multivariate levels (partial correlations and ratios). Pregnanolone is a neuroactive positive allosteric modulator (PAM) at Gamma-aminobutyric acid (GABA) receptors, enhancing synaptic plasticity and mitochondrial function. It also interacts with glutamatergic N-methyl-D-aspartate (NMDA) receptor, serotonin, and T-type calcium channels [55]. Imbalances in pregnanolone and allopregnanolone (pregnanolone being the precursor to allopregnanolone) have been linked to neuroinflammation, multiple sclerosis (MS), Alzheimer’s disease, Parkinson’s disease and other neuropsychiatric disorders ([56] for review). In ME/CFS, elevated levels of progesterone and its derivatives including pregnanolone and allopregnanolone, have been found in women compared to controls [57]. Neurosteroid brain disturbances align with core ME/CFS symptoms, including fatigue, cognitive dysfunction, orthostatic intolerance (baroreflex dysregulation), pain, and sleep disturbances. Further research, especially using cerebrospinal fluid (CSF) measurements, is needed to confirm these central nervous system findings and explore the roles of pregnanolone, allopregnanolone, and related neurosteroids in ME/CFS.

### Limitations

We acknowledge the exploratory sample size (*n* = 24 per group) and the current study’s cross-sectional nature which limits the ability to establish causality or determine the directionality of relationships among steroid hormones and ME/CFS. Longitudinal studies are needed to capture changes over time and provide insight into causal pathways. The study did not account for differences in duration of illness, physical activity levels, BMI, medication use (including hormonal contraceptives, hormone replacement therapy (HRT), intake of supplements, nor reproductive and menstrual cycle phase, all of which could affect steroid hormone levels. Controlling for these factors in future research would improve the precision and generalisability of the findings. In terms of the analytical approach, whilst UPSFC-MS/MS enables rapid, high-throughput profiling with strong chromatographic resolution, particularly for isomeric and keto-containing steroids through oxime derivatisation, it may have reduced sensitivity for very low-abundance analytes compared to GC-MS and does not capture the full spectrum of steroid metabolites. Complementary mass spectrometry approaches, including alternative ionisation or derivatisation strategies would be valuable to incorporate in future studies to support and extend these findings. Additionally, while acknowledging that more sophisticated methods, such as regularisation techniques (e.g. ridge regression partial correlation) may be better suited for handling high-dimensional data with multicollinearity, we chose spearman partial correlation due to the current sample size and the exploratory nature of this study. Despite these methodological limitations, the significant results obtained suggest disturbed steroid networks in ME/CFS and provides a valuable foundation for further research.

## Conclusions

This comprehensive exploratory workup underscores the urgent need for well controlled, longitudinal endocrine observational trials in the ME/CFS field. Such research will help reduce variability related to sample timing and cohort selection (e.g. time of day, menstrual cycle timing, and reproductive status) and capture the normal fluctuations and dynamics of the steroid network, thereby improving our understanding of endocrine pathophysiology in ME/CFS. Key recommendation for future studies includes larger cohorts, longitudinal sampling, menstrual cycle and diurnal aware sampling protocols, and functional follow-up studies to test enzyme or receptor density hypotheses. Furthermore, given the endocrine system’s wide-reaching influence across multiple physiological domains, including the immune system, metabolism, and the nervous system, a systems biology approach leveraging multi-omics technologies offers a powerful framework for exploring the interconnected pathways underlying ME/CFS. This approach would enable the integration of hormone data with immune, metabolic and neurological profiles to explore homeostatic disruptions, regulatory imbalances, and potential biomarker panels that may not be apparent when examining individual systems in isolation.

## Electronic supplementary material

Below is the link to the electronic supplementary material.

Supplementary Material 1

## Data Availability

The dataset analysed during the current study is available from the corresponding author on reasonable request.

## Abbreviations

11DOC: 11-deoxycortisol
17OHP: 17α-hydroxyprogesterone
ADT: Androsterone
AED: Androstenedione
Aldo: Aldosterone
B: Corticosterone
BH: Benjamini-hochberg
Cot: Cortisone
CTRL: Control
CSF: Cerebrospinal fluid
DHEA: Dehydroepiandrosterone
DOC: 1-deoxycorticosterone
E1: Estrone
E2: Estradiol
Etn: Etiocholanolone
F: Cortisol
FDR: False discovery rate
GABA: Gamma-aminobutyric acid
GC: Gas chromatography
HPA: Hypothalamic-pituitary-adrenal
HPG: Hypothalamic-pituitary-gonadal
IOM: Institute of medicine
LOD: Limit of detection
LLE: Liquid-liquid extraction
ME/CFS: Myalgic encephalomyelitis/chronic fatigue syndrome
MRM: Multiple reaction monitoring
MS: Mass spectrometry
MTBE: Methyl tert-butyl ether
NMDAR: N-methyl-D-aspartate receptor
OPLS-DA: Orthogonal partial least squares discriminant analysis
P4: Progesterone
P5: Pregnenolone
PEM: Post-exertional malaise
PLN or PNL: Pregnanolone
Q²: Cross-validated explained variance
R²X: Explained variance of predictors
R²Y: Explained variance of response
STD: Standard deviation
T: Testosterone
UPSFC-MS: Ultra-performance supercritical fluid chromatography–mass spectrometry
VIP: Variable importance in projection
WHO: World health organization
