# Supplementary material for: Steroid dynamics in myalgic encephalomyelitis / chronic fatigue syndrome: a case-control study using ultra performance supercritical fluid chromatography tandem mass spectrometry
Source: J Transl Med. 2025 Jul 25;23:829. doi: 10.1186/s12967-025-06841-4 (PMC12291238; doi:10.1186/s12967-025-06841-4)
Supplement: Supplementary file 1 — Supplementary Material 1 [file 12967_2025_6841_MOESM1_ESM.docx]

**Supplementary Material**

**Table 1A. Spearman Correlations Between Circulating Steroid Hormone Levels and Clinical Fatigue Severity Scores**

| Steroid | Clinical Rating | ρ (Spearman) | p value | Adjusted p value | n |
| --- | --- | --- | --- | --- | --- |
| Aldosterone | Mental Fatigue Scale | 0.4 | 0.11 | 0.48 | 17 |
| Aldosterone | Fibro Fatigue Scale | 0.29 | 0.24 | 0.67 | 18 |
| Androsterone | Mental Fatigue Scale | -0.32 | 0.2 | 0.52 | 18 |
| Androsterone | Fibro Fatigue Scale | 0.09 | 0.71 | 0.84 | 19 |
| Androstenedione | Mental Fatigue Scale | 0.03 | 0.89 | 0.89 | 18 |
| Androstenedione | Fibro Fatigue Scale | 0.38 | 0.11 | 0.67 | 19 |
| Cortisol | Mental Fatigue Scale | 0.35 | 0.15 | 0.48 | 18 |
| Cortisol | Fibro Fatigue Scale | 0.28 | 0.25 | 0.67 | 19 |
| Cortisone | Mental Fatigue Scale | 0.25 | 0.31 | 0.58 | 18 |
| Cortisone | Fibro Fatigue Scale | 0.39 | 0.1 | 0.67 | 19 |
| Corticosterone | Mental Fatigue Scale | 0.11 | 0.66 | 0.71 | 18 |
| Corticosterone | Fibro Fatigue Scale | 0.14 | 0.56 | 0.78 | 19 |
| 11-Deoxycorticosterone | Mental Fatigue Scale | 0.22 | 0.4 | 0.58 | 17 |
| 11-Deoxycorticosterone | Fibro Fatigue Scale | 0.34 | 0.17 | 0.67 | 18 |
| Dehydroepiandrosterone (DHEA) | Mental Fatigue Scale | -0.37 | 0.14 | 0.48 | 18 |
| Dehydroepiandrosterone (DHEA) | Fibro Fatigue Scale | 0.05 | 0.84 | 0.9 | 19 |
| Etiocholanolone | Mental Fatigue Scale | 0.14 | 0.59 | 0.7 | 18 |
| Etiocholanolone | Fibro Fatigue Scale | 0.13 | 0.58 | 0.78 | 19 |
| 17α-Hydroxyprogesterone | Mental Fatigue Scale | -0.22 | 0.38 | 0.58 | 18 |
| 17α-Hydroxyprogesterone | Fibro Fatigue Scale | 0.29 | 0.22 | 0.67 | 19 |
| Pregnenolone | Mental Fatigue Scale | -0.2 | 0.43 | 0.58 | 17 |
| Pregnenolone | Fibro Fatigue Scale | -0.09 | 0.73 | 0.84 | 18 |
| Pregnanolone | Mental Fatigue Scale | -0.26 | 0.3 | 0.58 | 18 |
| Pregnanolone | Fibro Fatigue Scale | -0.2 | 0.4 | 0.78 | 19 |
| Progesterone | Mental Fatigue Scale | 0.39 | 0.11 | 0.48 | 18 |
| Progesterone | Fibro Fatigue Scale | 0.2 | 0.4 | 0.78 | 19 |
| Testosterone | Mental Fatigue Scale | -0.39 | 0.12 | 0.48 | 17 |
| Testosterone | Fibro Fatigue Scale | -0.18 | 0.47 | 0.78 | 18 |
| Estrone | Mental Fatigue Scale | -0.25 | 0.33 | 0.58 | 18 |
| Estrone | Fibro Fatigue Scale | -0.15 | 0.54 | 0.78 | 19 |
| Estradiol | Mental Fatigue Scale | 0.13 | 0.61 | 0.7 | 18 |
| Estradiol | Fibro Fatigue Scale | 0.02 | 0.94 | 0.94 | 19 |

**Table 1B. Spearman Correlations Between Circulating Steroid Hormone Levels and Clinical Fatigue Severity Scores in Females**

| Steroid | Clinical Rating | ρ (Spearman) | p value | Adjusted p value | | n |
| --- | --- | --- | --- | --- | --- | --- |
| Aldosterone | Mental Fatigue Scale | 0.096 | 0.779 | | 0.909 | 11 |
| Aldosterone | Fibro Fatigue Scale | 0.102 | 0.753 | | 0.957 | 12 |
| Androsterone | Mental Fatigue Scale | 0.021 | 0.948 | | 0.948 | 12 |
| Androsterone | Fibro Fatigue Scale | 0.499 | 0.083 | | 0.872 | 13 |
| Androstenedione | Mental Fatigue Scale | -0.116 | 0.720 | | 0.909 | 12 |
| Androstenedione | Fibro Fatigue Scale | 0.424 | 0.149 | | 0.872 | 13 |
| Cortisol | Mental Fatigue Scale | -0.270 | 0.396 | | 0.791 | 12 |
| Cortisol | Fibro Fatigue Scale | -0.019 | 0.950 | | 0.957 | 13 |
| Cortisone | Mental Fatigue Scale | 0.225 | 0.483 | | 0.858 | 12 |
| Cortisone | Fibro Fatigue Scale | 0.284 | 0.347 | | 0.957 | 13 |
| Corticosterone | Mental Fatigue Scale | -0.442 | 0.150 | | 0.778 | 12 |
| Corticosterone | Fibro Fatigue Scale | -0.229 | 0.452 | | 0.957 | 13 |
| 11-Deoxycorticosterone | Mental Fatigue Scale | -0.064 | 0.852 | | 0.909 | 11 |
| 11-Deoxycorticosterone | Fibro Fatigue Scale | 0.151 | 0.640 | | 0.957 | 12 |
| Dehydroepiandrosterone (DHEA) | Mental Fatigue Scale | 0.063 | 0.845 | | 0.909 | 12 |
| Dehydroepiandrosterone (DHEA) | Fibro Fatigue Scale | 0.410 | 0.164 | | 0.872 | 13 |
| Etiocholanolone | Mental Fatigue Scale | 0.311 | 0.326 | | 0.791 | 12 |
| Etiocholanolone | Fibro Fatigue Scale | 0.127 | 0.679 | | 0.957 | 13 |
| 17α-Hydroxyprogesterone | Mental Fatigue Scale | -0.484 | 0.111 | | 0.778 | 12 |
| 17α-Hydroxyprogesterone | Fibro Fatigue Scale | 0.182 | 0.552 | | 0.957 | 13 |
| Pregnenolone | Mental Fatigue Scale | -0.079 | 0.806 | | 0.909 | 12 |
| Pregnenolone | Fibro Fatigue Scale | 0.024 | 0.939 | | 0.957 | 13 |
| Pregnanolone | Mental Fatigue Scale | -0.403 | 0.194 | | 0.778 | 12 |
| Pregnanolone | Fibro Fatigue Scale | -0.069 | 0.823 | | 0.957 | 13 |
| Progesterone | Mental Fatigue Scale | 0.326 | 0.301 | | 0.791 | 12 |
| Progesterone | Fibro Fatigue Scale | 0.132 | 0.667 | | 0.957 | 13 |
| Testosterone | Mental Fatigue Scale | 0.287 | 0.392 | | 0.791 | 11 |
| Testosterone | Fibro Fatigue Scale | 0.018 | 0.957 | | 0.957 | 12 |
| Estrone | Mental Fatigue Scale | -0.409 | 0.187 | | 0.778 | 12 |
| Estrone | Fibro Fatigue Scale | -0.175 | 0.568 | | 0.957 | 13 |
| Estradiol | Mental Fatigue Scale | -0.095 | 0.768 | | 0.909 | 12 |
| Estradiol | Fibro Fatigue Scale | -0.058 | 0.850 | | 0.957 | 13 |

**Table 1C. Spearman Correlations Between Circulating Steroid Hormone Levels and Clinical Fatigue Severity Scores in Males**

| Steroid | Clinical Rating | ρ (Spearman) | p value | Adjusted p value | | n |
| --- | --- | --- | --- | --- | --- | --- |
| Aldosterone | Mental Fatigue Scale | -0.232 | 0.658 | | 0.945 | 6 |
| Aldosterone | Fibro Fatigue Scale | 0.486 | 0.356 | | 0.813 | 6 |
| Androsterone | Mental Fatigue Scale | 0.116 | 0.827 | | 0.945 | 6 |
| Androsterone | Fibro Fatigue Scale | -0.371 | 0.497 | | 0.861 | 6 |
| Androstenedione | Mental Fatigue Scale | 0.261 | 0.618 | | 0.945 | 6 |
| Androstenedione | Fibro Fatigue Scale | 0.371 | 0.497 | | 0.861 | 6 |
| Cortisol | Mental Fatigue Scale | 0.232 | 0.658 | | 0.945 | 6 |
| Cortisol | Fibro Fatigue Scale | 0.829 | 0.058 | | 0.187 | 6 |
| Cortisone | Mental Fatigue Scale | 0.899 | 0.015 | | 0.238 | 6 |
| Cortisone | Fibro Fatigue Scale | 0.829 | 0.058 | | 0.187 | 6 |
| Corticosterone | Mental Fatigue Scale | 0.841 | 0.036 | | 0.266 | 6 |
| Corticosterone | Fibro Fatigue Scale | 0.886 | 0.033 | | 0.187 | 6 |
| 11-Deoxycorticosterone | Mental Fatigue Scale | 0.812 | 0.050 | | 0.266 | 6 |
| 11-Deoxycorticosterone | Fibro Fatigue Scale | 0.886 | 0.033 | | 0.187 | 6 |
| Dehydroepiandrosterone (DHEA) | Mental Fatigue Scale | -0.116 | 0.827 | | 0.945 | 6 |
| Dehydroepiandrosterone (DHEA) | Fibro Fatigue Scale | -0.143 | 0.803 | | 0.917 | 6 |
| Etiocholanolone | Mental Fatigue Scale | 0.618 | 0.191 | | 0.510 | 6 |
| Etiocholanolone | Fibro Fatigue Scale | 0.319 | 0.538 | | 0.861 | 6 |
| 17α-Hydroxyprogesterone | Mental Fatigue Scale | 0.754 | 0.084 | | 0.334 | 6 |
| 17α-Hydroxyprogesterone | Fibro Fatigue Scale | 0.829 | 0.058 | | 0.187 | 6 |
| Pregnenolone | Mental Fatigue Scale | 0.000 | 1.000 | | 1.000 | 5 |
| Pregnenolone | Fibro Fatigue Scale | -0.205 | 0.741 | | 0.917 | 5 |
| Pregnanolone | Mental Fatigue Scale | -0.154 | 0.771 | | 0.945 | 6 |
| Pregnanolone | Fibro Fatigue Scale | -0.698 | 0.123 | | 0.328 | 6 |
| Progesterone | Mental Fatigue Scale | -0.493 | 0.321 | | 0.708 | 6 |
| Progesterone | Fibro Fatigue Scale | 0.143 | 0.803 | | 0.917 | 6 |
| Testosterone | Mental Fatigue Scale | 0.667 | 0.148 | | 0.474 | 6 |
| Testosterone | Fibro Fatigue Scale | 0.257 | 0.658 | | 0.917 | 6 |
| Estrone | Mental Fatigue Scale | 0.000 | 1.000 | | 1.000 | 6 |
| Estrone | Fibro Fatigue Scale | 0.029 | 1.000 | | 1.000 | 6 |
| Estradiol | Mental Fatigue Scale | 0.464 | 0.354 | | 0.708 | 6 |
| Estradiol | Fibro Fatigue Scale | -0.086 | 0.919 | | 0.981 | 6 |

**Table 2. Comparison of absolute steroid levels between Myalgic Encephalomyelitis/ Chronic Fatigue Syndrome and Control groups**

| **STEROID** | **[CTRL] (mean)** | **[ME/CFS] (mean)** | **[CTRL] (std)** | **[ME/CFS] (std)** | **p value** | **Cohen’s d** | **Estimated sample size required to detect effect size (Cohen’s d)** |
| --- | --- | --- | --- | --- | --- | --- | --- |
| **Aldosterone (Aldo)** | 0.357 | 0.296 | 0.300 | 0.209 | 0.516 | -0.236 | 282.8 |
| **Androsterone (ADT)** | 5.957 | 9.495 | 4.719 | 11.691 | 0.483 | 0.397 | 100.6 |
| **Androstenedione (AED)** | 0.773 | 1.072 | 0.429 | 1.133 | 0.509 | 0.349 | 129.8 |
| **Cortisol (F)** | 138.064 | 135.157 | 62.304 | 61.887 | 0.718 | -0.047 | 7107.2 |
| **Cortisone (Cot)** | 26.504 | 25.189 | 8.562 | 11.179 | 0.415 | -0.132 | 901.9 |
| **Corticosterone (B)** | 2.970 | 3.000 | 3.202 | 2.806 | 0.829 | 0.01 | 156978.2 |
| **1-deoxycorticosterone (DOC)** | 0.324 | 0.252 | 0.475 | 0.386 | 0.818 | -0.166 | 570.6 |
| **11-deoxycortisol (11DOC)** | 0.708 | 0.509 | 0.840 | 0.516 | 0.591 | -0.285 | 194.2 |
| **Dehydroepiandrosterone (DHEA)** | 20.713 | 27.484 | 26.800 | 31.127 | 0.284 | 0.233 | 290.1 |
| **Etiocholanolone (Etn)** | 2.433 | 2.322 | 2.467 | 2.295 | 0.992 | -0.047 | 7107.2 |
| **17α-hydroxyprogesterone (17OHP)** | 0.381 | 0.389 | 0.207 | 0.361 | 0.332 | 0.027 | 21534.2 |
| **Pregnanolone (PNL)** | 1.695 | 0.644 | 2.332 | 1.218 | 0.095 | -0.565 | 50.2 |
| **Pregnenolone (P_5)_** | 4.131 | 4.152 | 8.919 | 5.265 | 0.327 | 0.003 | 1744192.2 |
| **Progesterone (P_4)_** | 0.477 | 1.473 | 0.193 | 3.295 | 0.642 | 0.427 | 87.1 |
| **Testosterone (T)** | 1.694 | 1.892 | 2.193 | 2.873 | 0.837 | 0.077 | 2648.6 |
| **Estrone (E1)*** | 82.727 | 45.340 | 167.791 | 39.091 | 0.876 | -0.307 | 167.5 |
| **Estradiol (E2)*** | 30.254 | 41.060 | 30.420 | 67.805 | 0.445 | 0.206 | 370.9 |

*Abbreviations:* Aldo; aldosterone, ADT; androsterone, AED; androstenedione, F; cortisol, Cot; cortisone, B; corticosterone, DOC; 1-deoxycorticosterone, 11DOC; 11-deoxycortisol, DHEA; dehydroepiandrosterone, Etn; etiocholanolone, 17OHP; 17α-hydroxyprogesterone, PNL; pregnanolone, P_5_; pregnenolone, P_4_; progesterone, T; testosterone, E1; estrone, E2; estradiol. * ng/mol

**Table 2. ME/CFS and Control comparison in absolute steroid levels in females.**

| **Steroid** | **CTRL (mean)** | **ME/CFS (mean)** | **CTRL (STD)** | **ME/ CFS (STD)** | **p value** | **Cohen’s d** | **Estimated sample size required to detect effect size (Cohen’s d)** |
| --- | --- | --- | --- | --- | --- | --- | --- |
| **Aldosterone (Aldo)** | 0.316 | 0.324 | 0.175 | 0.212 | 0.940 | 0.041 | 9339.3 |
| **Androsterone (ADT)** | 4.708 | 5.837 | 4.417 | 5.983 | 0.763 | 0.215 | 340.6 |
| **Androstenedione (AED)** | 0.750 | 1.218 | 0.491 | 1.369 | 0.291 | 0.455 | 76.8 |
| **Cortisol (F)** | 139.623 | 153.090 | 59.494 | 59.510 | 0.440 | 0.226 | 308.3 |
| **Cortisone (Cot)** | 26.581 | 25.168 | 8.450 | 11.536 | 0.407 | -0.140 | 801.9 |
| **Corticosterone (B)** | 2.683 | 3.624 | 2.035 | 3.185 | 0.318 | 0.352 | 127.7 |
| **11-Deoxycorticosterone (DOC)** | 0.216 | 0.140 | 0.416 | 0.163 | 0.911 | -0.241 | 271.2 |
| **11-Deoxycortisol (11DOC)** | 0.703 | 0.534 | 0.885 | 0.549 | 0.880 | -0.229 | 300.3 |
| **Dehydroepiandrosterone (DHEA)** | 10.197 | 13.411 | 6.556 | 10.035 | 0.346 | 0.379 | 110.3 |
| **Etiocholanolone (Etn)** | 1.848 | 1.863 | 2.440 | 1.928 | 0.608 | 0.007 | 320362.6 |
| **17α-Hydroxyprogesterone (17OHP)** | 0.296 | 0.345 | 0.186 | 0.348 | 0.692 | 0.176 | 507.7 |
| **Pregnanolone (PNL)** | 1.714 | 0.624 | 2.650 | 1.350 | 0.205 | -0.518 | 59.5 |
| **Pregnenolone (P5)** | 4.136 | 4.205 | 10.392 | 5.227 | 0.297 | 0.008 | 245277.9 |
| **Progesterone (P4)** | 0.508 | 1.994 | 0.211 | 3.971 | 0.692 | 0.528 | 57.3 |
| **Testosterone (T)** | 0.313 | 0.388 | 0.137 | 0.261 | 0.678 | 0.360 | 122.1 |
| **Estrone (E1)*** | 103.031 | 47.616 | 204.172 | 44.784 | 1.000 | -0.375 | 112.6 |
| **Estradiol (E2)*** | 38.288 | 48.366 | 34.119 | 78.934 | 0.415 | 0.166 | 570.6 |

*Abbreviations:* ME/CFS; Myalgic Encephalomyelitis/ Chronic Fatigue Syndrome, STD; standard deviation, Steroid concentrations are reported in nmol/mL (pmol/mL for estrone* and estradiol*).

**Table 3. Absolute steroid level comparison between Myalgic Encephalomyelitis/ Chronic Fatigue Syndrome and Control Groups in males.**

| **Steroid** | **CTRL (mean)** | **ME/CFS (mean)** | **CTRL (STD)** | **ME/ CFS (STD)** | **p value** | **Cohen’s d** | **Estimated sample size required to detect effect size (Cohen’s d)** |
| --- | --- | --- | --- | --- | --- | --- | --- |
| **Aldosterone (Aldo)** | 0.440 | 0.240 | 0.467 | 0.202 | 0.227 | -0.556 | 51.8 |
| **Androsterone (ADT)** | 8.454 | 16.810 | 4.549 | 16.753 | 0.189 | 0.681 | 34.8 |
| **Androstenedione (AED)** | 0.819 | 0.779 | 0.292 | 0.243 | 0.713 | -0.149 | 708.0 |
| **Cortisol (F)** | 134.946 | 99.290 | 71.784 | 52.822 | 0.189 | -0.566 | 50.0 |
| **Cortisone (Cot)** | 26.350 | 25.230 | 9.373 | 11.201 | 0.431 | -0.108 | 1346.8 |
| **Corticosterone (B)** | 3.544 | 1.753 | 4.926 | 1.207 | 0.564 | -0.499 | 64.0 |
| **11-Deoxycorticosterone (DOC)** | 0.540 | 0.475 | 0.539 | 0.590 | 0.784 | -0.115 | 1187.9 |
| **11-Deoxycortisol (11DOC)** | 0.719 | 0.459 | 0.801 | 0.473 | 0.493 | -0.395 | 101.6 |
| **Dehydroepiandrosterone (DHEA)** | 41.745 | 55.629 | 38.854 | 40.122 | 0.156 | 0.352 | 127.7 |
| **Etiocholanolone (Etn)** | 3.603 | 3.240 | 2.213 | 2.810 | 0.792 | -0.144 | 758.0 |
| **17α-Hydroxyprogesterone (17OHP)** | 0.551 | 0.476 | 0.127 | 0.395 | 0.226 | -0.256 | 240.5 |
| **Pregnanolone (PNL)** | 1.656 | 0.684 | 1.681 | 0.982 | 0.217 | -0.706 | 32.5 |
| **Pregnenolone(P5)** | 4.123 | 4.045 | 5.474 | 5.703 | 0.956 | -0.014 | 80091.4 |
| **Progesterone (P4)** | 0.415 | 0.431 | 0.140 | 0.120 | 0.916 | 0.123 | 1038.6 |
| **Testosterone (T)** | 4.455 | 4.901 | 1.640 | 3.385 | 0.958 | 0.168 | 557.1 |
| **Estrone (E1)** | 42.119 | 40.788 | 18.724 | 26.226 | 0.792 | -0.058 | 4667.3 |
| **Estradiol (E2)** | 14.188 | 26.450 | 10.175 | 37.266 | 0.916 | 0.449 | 78.8 |

*Abbreviations:* ME/CFS; Myalgic Encephalomyelitis/ Chronic Fatigue Syndrome, STD; standard deviation, Steroid concentrations are reported in nmol/mL (pmol/mL for estrone* and estradiol*)
